# Logarithmic sensing in *Bacillus subtilis* aerotaxis

**DOI:** 10.1038/npjsba.2016.36

**Published:** 2017-01-19

**Authors:** Filippo Menolascina, Roberto Rusconi, Vicente I Fernandez, Steven Smriga, Zahra Aminzare, Eduardo D Sontag, Roman Stocker

**Affiliations:** 1 Institute for Bioengineering, School of Engineering, The University of Edinburgh, Scotland, UK; 2 SynthSys—Centre for Synthetic and Systems Biology, The University of Edinburgh, Scotland, UK; 3 Ralph M Parsons Laboratory, Department of Civil and Environmental Engineering, Massachusetts Institute of Technology, Cambridge, MA, USA; 4 Institute of Environmental Engineering, Department of Civil, Environmental and Geomatic Engineering, Zurich, Switzerland; 5 The Program in Applied and Computational Mathematics, Princeton, NJ, USA; 6 Department of Mathematics, Hill Center Rutgers, The State University of New Jersey, Piscataway, NJ, USA

## Abstract

Aerotaxis, the directed migration along oxygen gradients, allows many microorganisms to locate favorable oxygen concentrations. Despite oxygen’s fundamental role for life, even key aspects of aerotaxis remain poorly understood. In *Bacillus subtilis,* for example, there is conflicting evidence of whether migration occurs to the maximal oxygen concentration available or to an optimal intermediate one, and how aerotaxis can be maintained over a broad range of conditions. Using precisely controlled oxygen gradients in a microfluidic device, spanning the full spectrum of conditions from quasi-anoxic to oxic (60 n mol/l–1 m mol/l), we resolved *B. subtilis’* ‘oxygen preference conundrum’ by demonstrating consistent migration towards maximum oxygen concentrations (‘monotonic aerotaxis’). Surprisingly, the strength of aerotaxis was largely unchanged over three decades in oxygen concentration (131 n mol/l–196 μ mol/l). We discovered that in this range *B. subtilis* responds to the logarithm of the oxygen concentration gradient, a rescaling strategy called ‘log-sensing’ that affords organisms high sensitivity over a wide range of conditions. In these experiments, high-throughput single-cell imaging yielded the best signal-to-noise ratio of any microbial taxis study to date, enabling the robust identification of the first mathematical model for aerotaxis among a broad class of alternative models. The model passed the stringent test of predicting the transient aerotactic response despite being developed on steady-state data, and quantitatively captures both monotonic aerotaxis and log-sensing. Taken together, these results shed new light on the oxygen-seeking capabilities of *B. subtilis* and provide a blueprint for the quantitative investigation of the many other forms of microbial taxis.

## Introduction

Oxygen mediates the conversion of carbon sources to cellular energy and is the most common electron acceptor used in cellular respiration. In order to locate optimal oxygen conditions in their environment, several species of bacteria have evolved the ability to sense and migrate along oxygen gradients, a strategy called aerotaxis.^1^ Aerotaxis is fundamental to many ecological processes. Within microbial mats at the sediment–water interface, the filamentous bacteria *Beggiatoa* use aerotaxis to glide along steep vertical oxygen gradients toward preferred micro-oxic depths.^2^ The sulfur-reducing bacteria *Desulfovibrio* swim to accumulate in regions of specific, low oxygen concentration (~500 n mol/l or 0.04%), where conditions are thermodynamically favorable for anaerobic respiration.^3^ An important phytopathogenic bacterium, *Ralstonia solanacearum*, uses aerotaxis to attack the roots of its plant hosts, including tomato and banana plants, causing their wilting and death.^4^ *Escherichia coli,* a common inhabitant of the lower digestive tract of warm blooded animals, has been found to exploit aerotaxis to cross the mucosal layer protecting epithelial surface in the intestine and expedite its colonization.^5^ More recently, *Caulobacter crescentus*, a monotrichous bacterium found in aquatic environments, has been observed to perform aerotaxis, adjusting its motility based on a dynamic rescaling of oxygen gradients.^6^ Besides its role in energy harvesting, aerotaxis is involved in collective bacterial migrations, including bioconvection, where bacteria that swim up oxygen gradients accumulate and render water denser, causing convection and mixing.^7,8^

Aerotaxis was the first of all microbial taxis behaviors (i.e., directional migration responses to external stimuli) to be described, when in 1881 Theodor Engelmann observed bacteria moving towards the chloroplasts of algae in response to the oxygen produced by photosynthesis.^9^ Despite its early discovery, however, our understanding of aerotaxis has remained rather poor and mostly qualitative. For example, even for the model bacterium *Bacillus subtilis* it has remained unclear whether cells seek an optimal, intermediate oxygen concentration (e.g., 200 μ mol/l or *~*15%;^1,10^ percentage values are based on oxygen saturation in water under laboratory conditions (temperature 25 °C and pressure 100 kPa)) or swim towards the highest oxygen concentration available (1.3 m mol/l or 100%),^11^ a strategy with potentially detrimental physiological effects.^12^ Beyond this ‘oxygen preference conundrum’, even the obligately aerobic nature of *B. subtilis*,^13^ once believed to be a robust trait of this organism, has recently been put to question by results documenting its anaerobic growth.^14^ This lack of understanding of concentration preferences in aerotaxis, in turn, prevents quantitative predictions of population dynamics in oxygen gradients.

Sensing mechanisms for aerotaxis fall in two categories. A first mechanism is based on the sensing of the intracellular energy status to determine the need for additional oxygen. This mechanism belongs to the class of energy-tactic behaviors,^1^ is found in *E. coli*, *Azospirillum brasilense, Salmonella tiphymurium* and *Pseudomonas aeruginosa*, and has received considerable attention.^15,16^ In *E. coli*, aerotaxis is mediated by the receptors *Aer* and *Tsr*. The former has recently been proposed to monitor the cell’s metabolic state by gauging the activity of the electron transport system,^17^ whereas *Tsr* has been speculated to sense the proton-motive force, a measure of the potential energy stored in the cell.^18^ A second mechanism for aerotaxis is based on the sensing of extracellular oxygen via its direct binding to heme-containing receptors like *HemAT*. This mechanism, independent of metabolism and similar to classic chemotaxis,^19^ is found for example in *B. subtilis* and *Halobacterium salinarum*, and has received limited attention to date.^6,19^ For both forms of aerotaxis, there are no quantitative models of the cellular response to oxygen gradients.

Here we present a high-resolution experimental characterization of aerotaxis, focusing on the case of direct oxygen sensing in *B. subtilis*, a gram positive bacterium that is widespread in a broad range of environments.^12^ The robustness of the data and the breadth of conditions examined allowed us to identify a quantitative population model for aerotaxis in *B. subtilis* and to resolve the oxygen preference conundrum for this bacterium by demonstrating that cells seek the highest oxygen concentration available under the full spectrum of conditions tested.

Traditional techniques to study aerotaxis have only enabled very limited quantification of this migration strategy. Observations of bacterial populations in capillaries, sealed at one end and exposed to a controlled oxygen concentration at the other end, revealed the formation of bacterial bands.^1^ These bands have been interpreted as evidence for preferred oxygen concentrations by bacteria, yet conclusive interpretation and a quantitative analysis have remained difficult, because oxygen gradients are governed by both diffusion and respiration and thus poorly quantifiable with this approach. Oxygen measurements with microelectrodes^20^ partially addressed this problem but have the drawback of being invasive, altering the distribution of both bacteria and oxygen. In contrast, initial applications of microfluidic devices to chemotaxis^21^ and aerotaxis^6,16^ have demonstrated the potential of this approach to control gradients for taxis studies, while simultaneously visualizing population responses.

Here we used a new microfluidic device and computer-controlled gas mixers to generate precise, linear oxygen concentration profiles over oxygen conditions ranging from anoxic to oxic, and we applied video microscopy and image analysis to accurately quantify the response of extremely large numbers of individual cell coordinates. We observed that *B. subtilis* always swims towards the highest available oxygen concentration and, notably, displays the same magnitude of aerotaxis over oxygen gradients (∇*C*) spanning three orders of magnitude when the relative gradient (∇*C/C*) is conserved, indicating this bacterium rescales its response to oxygen gradients via logarithmic sensing. We show that the vastly improved level of environmental control and data robustness of this approach over traditional ones permits the identification of a predictive mathematical model of aerotaxis in *B. subtilis* that captures logarithmic sensing and provides a blueprint for the quantitative study of other forms of microbial taxis.

## Results and Discussion

**Figure 1:**
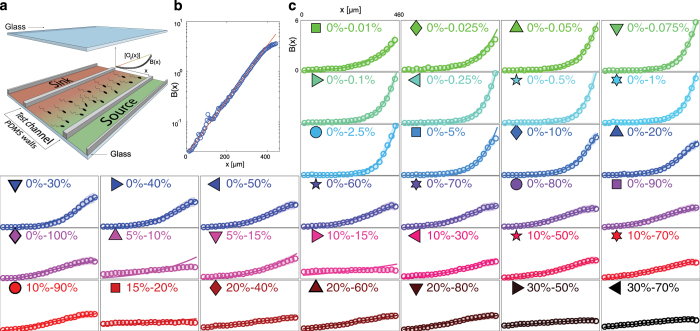
Aerotactic response of *Bacillus subtilis*. (**a**) The microfluidic device used to probe aerotaxis in *B. subtilis* consists of three parallel channels fabricated in polydimethylsiloxane (PDMS), with top and bottom glass surfaces. Flowing oxygen at prescribed concentrations in the source (green) and sink (red) channels created a linear oxygen concentration profile ([O_2_(*x*)]) in the test channel, where the bacterial response was monitored by video microscopy. (**b**) Steady-state distribution of bacteria across the test channel, *B*(*x*) (blue circles), for the 0–20% gradient (sink concentration—source concentration), along with the best exponential fit (red). (**c**) Steady-state aerotactic response, *B*(*x*), for all 33 oxygen conditions tested. For each panel, the *x* axis represents the test channel width (0<*x*<460 μm) and the *y* axis represents *B*(*x*) (0<*B*(*x*)<6). A uniform bacterial distribution would correspond to *B*(*x*)=1. Open circles are experimental data, thin solid lines are model predictions, the shaded envelope represents plus/minus one standard deviation on the predictions. The oxygen concentrations in the sink and source channels are reported for each condition and a symbol is assigned to each condition for reference in subsequent figures.

To study the aerotactic response of *B. subtilis* we exposed the bacteria to linear concentration profiles of oxygen across a microchannel and quantified the spatial distribution of cells along the width of the channel, *B*(*x*), at steady state. We devised a new microfluidic device made of polydimethylsiloxane (PDMS) featuring three parallel channels sealed by glass slides on the top and bottom (Figure 1a). The central ‘test’ channel hosts the cells, whereas the flanking ‘oxygen control’ channels each carry a flow of oxygen at a prescribed concentration, higher in the ‘source’ channel and lower in the ‘sink’ channel (Figure 1a). Oxygen diffuses from the source channel to the sink channel through the PDMS (which is oxygen permeable^22^) and the test channel, where it thereby forms a gradient. The two glass slides, being impermeable to gas, force oxygen diffusion to occur only sideways, which leads to a linear oxygen profile. This provides less flexibility in setting up arbitrary gradients, compared with prior approaches,^16^ but has the advantage of extreme fabrication simplicity (single-layer, two oxygen boundary conditions). A mathematical model of oxygen diffusion in this system (implemented in COMSOL Multiphysics 4.4; see ‘Derivation and identification of the mathematical model’ in Supplementary Materials and Methods), which also accounts for the top and bottom glass surfaces that are impermeable to oxygen, provides a quantitative prediction of the oxygen concentration and gradient that bacteria experience at every position in the test channel and confirms that the oxygen concentration profile is linear (Supplementary Figure S1). The model also shows that cells in the device are exposed to >90% of the total oxygen gradient between sink and source channels (see ‘Oxygen diffusion within the device’ and Supplementary Table S1).

To quantify aerotaxis we probed *B. subtilis*’ response at steady state in 33 different gradients, spanning four decades in gradient magnitude (from 0.26 n mol/l/μm to 2.56 μ mol/l/μm) and ranging from 0%–0.01% to 0%–100%. Experiments therefore covered a large spectrum of the oxygen conditions that *B. subtilis* may experience in nature, from quasi-anoxic to fully oxygenated.

**Figure 2:**
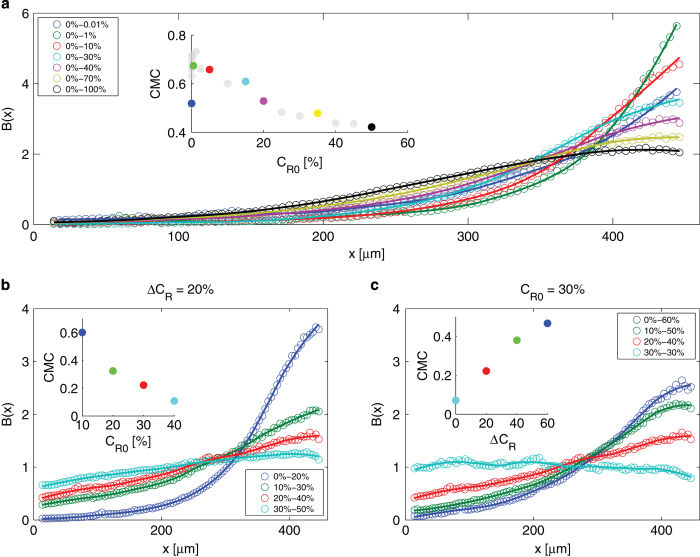
Oxygen preference in *B. subtilis* aerotaxis. (**a**) Normalized bacterial density profiles across the width of the test channel, *B*(*x*), for a subset of the experiments of the form 0%–*X*% (sink concentration—source concentration). Open circles are experimental data, solid lines are a smoothed version of the same data obtained with a Savitzky–Golay filter. Inset: The Chemotactic Migration Coefficient (CMC) for the same experiments, as a function of the oxygen concentration *C*_*R*0_ at mid-channel (*x*=230 μm). Color-coding of the data in the inset corresponds to the main panel, whereas gray circles are all other experiments of the form 0%–*X*%. (**b**) Aerotactic response, *B*(*x*), in experiments in which the oxygen gradient was kept constant (Δ*C*_*R*_=20% between source and sink channels) and the absolute oxygen concentration *C*_*R*0_ was varied. Inset: the CMC decreases with increasing absolute oxygen concentration. (**c**) Aerotactic response, *B*(*x*), in experiments in which the oxygen gradient (i.e., Δ*C*_*R*_) was varied and the absolute oxygen concentration C_*R*0_ was kept constant. Inset: the CMC increases with increasing oxygen gradient.

For all conditions tested, *B. subtilis* moved towards the highest oxygen concentration available (Figure 1c). Accumulation profiles *B*(*x*) were mostly exponentially shaped (Figure 1b). Positive aerotaxis was observed even when the highest oxygen concentration in the test channel was near saturation (0%–100% gradient, Figure 1c). In contrast to previous reports of a preferred oxygen concentration of 200 μ mol/l (i.e., ~15%) for *B. subtilis,*^10^ we consistently (19 experiments, Figure 1c) observed accumulation of cells to higher concentrations than 200 μ mol/l (although we did observe a decrease in the accumulation strength for oxygen concentrations above 20%; Figure 2a). These findings resolve the oxygen preference conundrum in *B. subtilis* and demonstrate that these bacteria always swim towards the highest available oxygen concentration.

Aerotaxis was strongest at very low oxygen concentrations, in particular in the micromolar regime (Figure 1c). For example, the concentration of bacteria reached a value six-fold higher than the mean near the top of the 0%–1% (0–13 μ mol/l) gradient (Figure 2a). To assess the strength of accumulation of bacteria, we calculated the Chemotactic Migration Coefficient (CMC), a metric frequently used in the chemotaxis literature^21^ that quantifies the distance that cells travel from the middle of the channel towards high chemoattractant concentrations (Figure 2a, inset). Focusing on gradients with 0% oxygen in the sink channel (Figure 2a), the strength of accumulation (CMC) showed a non-monotonic dependence on the mean oxygen concentration, *C*_R0_ (equivalently, the oxygen concentration at mid-channel, *x*=230 μm). The CMC increased with *C*_R0_ for gradients going from 0%–0.01% (0.015%/mm) to 0%–1% (1.5%/mm) and decreased for gradients larger than 0%–1% (Figure 2a, inset).

The magnitude of the aerotactic response depended on both the strength of the gradient, ∇*C* (=Δ*C*_R_/W, where Δ*C*_R_ is the difference between source and sink concentrations and W is the width of the channel), and the mean concentration of oxygen in the test channel, *C*_R0_ (Figure 1c). To tease apart the contribution of these two factors, we carried out two sets of experiments. In the first set, the oxygen gradient was fixed (Δ*C*_R_=20%) and we varied the mean oxygen concentration (*C*_R0_=10% to 40%). These experiments indicate that the strength of accumulation, CMC, decreases with increasing mean concentration (Figure 2b). In the second set of experiments, the mean oxygen concentration was fixed (*C*_R0_=30%) and we varied the oxygen gradient (Δ*C*_R_=0% to 60%, i.e., from 30%–30% to 0%–60%). These experiments show that the CMC increases with the oxygen gradient (Figure 2c). Taken together, these two sets of results led us to hypothesize that *B. subtilis* senses the relative gradient of oxygen, ∇*C/C*, or equivalently the gradient of the logarithm of oxygen concentration, ∇log*C*.

**Figure 3:**
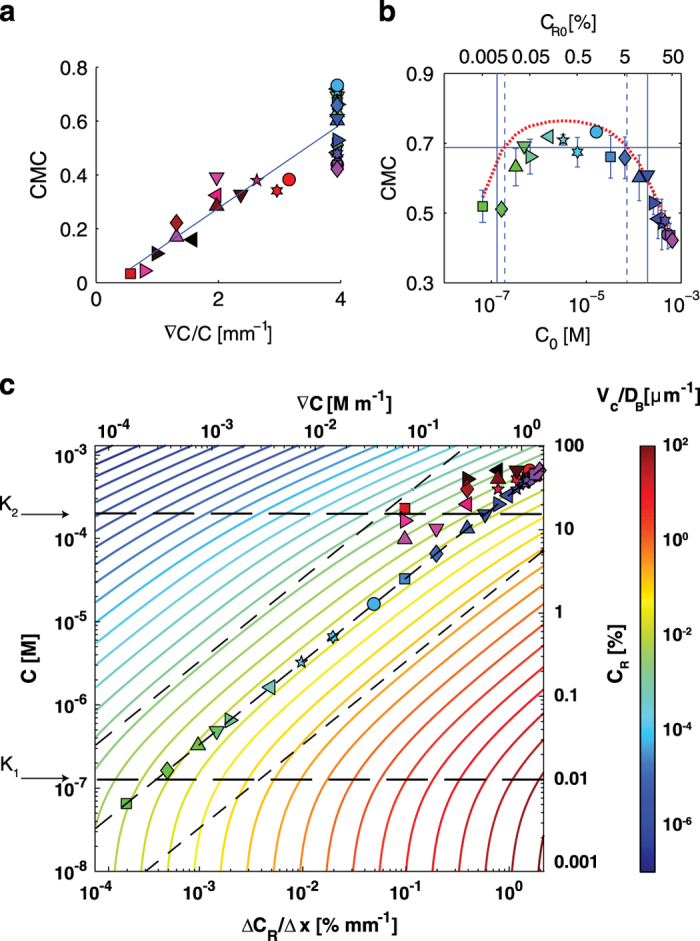
Logarithmic sensing in *B. subtilis* aerotaxis. (**a**) The Chemotactic Migration Coefficient (CMC) as a function of the relative gradient ∇*C/C*. The solid line represents the best linear fit to the data. (**b**) The CMC for the experiments of the type 0%–*X*%, with *X* ranging from 0.01 to 100: all experiments in this set have the same oxygen gradient ∇*C/C*, but different absolute concentrations *C*_*R*0_ (mid-channel concentration). Error bars represent s.e. calculated over the biological replicates (see Supplementary Table S1). The red dotted line is the CMC predicted by the model. The horizontal line denotes 90% of the maximum accumulation. Vertical solid lines represent *K*_1_ and *K*_2_ (equation (2)) as identified by the multi-experimental fitting procedure. Dashed blue lines delimit the region within which aerotactic accumulation was between 90% and 100% of the maximum. (**c**) Magnitude of the aerotactic response predicted by the model (equations (1 and 2)) over a broad range of oxygen concentrations *C* and concentration gradients ∇*C*, quantified in terms of the inverse of the accumulation length scale *Vc/D*_*B*_. The measured bacteria diffusivity *D*_*B*_ was used (Supplementary Figure S2). Black dashed lines denote *K*_1_ and *K*_2_ and delimit the logarithmic sensing regime. Symbols correspond to experimental conditions and are as defined in Figure 1c).

To test the hypothesis of logarithmic sensing, we computed the CMC for all experiments and studied its dependence on ∇*C*/*C*. In this expression we used the mid-channel concentration *C*_0_ for *C*, whereas ∇*C* is the same across the entire test channel. Results show that the CMC values over the entire set of 33 experiments collapse on a straight line when plotted against ∇*C*/*C* (Figure 3a), indicating that logarithmic-sensing underpins aerotaxis in *B. subtilis*. Evidence of logarithmic sensing in bacteria has to date been limited to the chemotaxis of *E. coli* to aspartate and serine,^23^ where it was proposed to originate in methylation-mediated adaptation. For *B. subtilis*, our limited understanding of the intracellular dynamics underlying aerotaxis prevents us from speculating on the molecular underpinnings of logarithmic sensing, but the ability of the bacteria to rescale their aerotactic response in this manner and retain high sensitivity over a broad range of conditions indicates that aerotaxis is an important and fine-tuned behavioral response for this organism. The spread observed at ∇*C*/*C*=4/mm (Figure 3a) allows to identify the end of the logarithmic sensing regime, i.e., the maximum accumulation strength (i.e., higher CMC) is only observed for oxygen concentrations *C*_R0_ up to 1% (Figure 2a, inset and central plateau in Figure 3b) and rapidly decreases at higher concentrations.

Logarithmic sensing applies over a wide range of oxygen concentrations. To see this, we consider all experiments of the type 0%–*X*%, with *X* comprised between 0.01 and 100, which all have the same value of ∇*C*/*C*=4/mm (because both ∇*C* and *C* are proportional to *X*). The dependence of CMC for these experiments on the mid-channel concentration *C*_0_ (Figure 3b) reveals a wide plateau ranging from *C*_0_=0.19 μ mol/l to 73.0 μ mol/l where the CMC values are all within ±7% of the mean CMC computed over this range (0.68). Taken together these results indicate that the magnitude of aerotaxis remains nearly unchanged over several orders of magnitude in oxygen concentration, provided ∇*C*/*C* is unchanged.

A mathematical model of aerotaxis, informed by known elements of the biophysics of *HemAT*-mediated oxygen sensing, successfully predicts both the existence of logarithmic sensing and the concentration range over which it applies. Following an approach developed for state-driven stochastic processes with re-orientations,^24^ we developed a one-dimensional advection diffusion model to predict the concentration of bacteria B(*x*,*t*), as

(1)∂B∂t=∂∂x(DB∂B∂x−VC(C,∇C)B)

where *D*_B_ is the diffusivity of the bacteria and *V*_C_ their chemotactic velocity. Analysis of cell trajectories in uniform oxygen concentrations showed that the diffusivity, which results from the random component of motility, is nearly constant between oxygen concentrations of 390 μ mol/l and 1.3 m mol/l, and diminishes below 26 μ mol/l (Supplementary Figure S2; see Supplementary Information for an in-depth analysis). This leaves only the chemotactic velocity *V*_C_ to be determined in order to have a complete model of aerotaxis.

The fundamental element of the aerotaxis model is the functional dependence of *V*_C_ on the oxygen concentration *C* and concentration gradient ∇*C*. Although many different functional forms of *V*_C_ have been proposed, they nearly all fall in three categories: Keller–Segel models (KS), where *V*_C_=*χ*_0_∇*C*/*C* and *χ*_0_ is the chemotactic sensitivity coefficient; Lapidus–Schiller models (LS), where *V*_C_=*χ*_0_/(*K*+*C*)^2^ and *K* is the chemoreceptor-ligand dissociation constant; and Rivero–Tranquillo–Buettner–Lauffenburger models (RTBL), where $V_{C}=\frac{2}{3}V\;\mathrm{tan h}\left(\frac{{K\chi }_{0}}{2V}\nabla C/{\left(K+C\right)}^{2}\right)$ and *V* is swimming speed. Interestingly, although all these models have been developed to study chemotaxis in *E. coli*, they all fail to capture an important feature of chemotaxis in this microorganism: logarithmic sensing over a finite range of concentrations.^23^ KS predicts logarithmic sensing (i.e., rescaling *C* by a constant leaves *V*_C_ unchanged) for any oxygen concentration, whereas neither LS nor RTBL support logarithmic sensing for any concentration.

We propose a new model for *V*_C_ that captures logarithmic sensing in *B. subtilis’* aerotaxis over a finite range of oxygen concentrations (Materials and Methods). We started from a one-dimensional Fokker-Planck model^24,25^ to capture the temporal evolution of the spatial distribution of bacteria in an oxygen gradient (Supplementary Information, Supplementary Equation (5)), modeling the exploration of the one-dimensional domain as a velocity jump process, where bacteria can either run with constant speed in the positive or negative *x* direction, or tumble (i.e., instantaneously change direction). The probability of tumbling is controlled by an intracellular variable, the receptor methylation state, which in turn depends on the extracellular oxygen concentration (Supplementary Information, Supplementary Equation (54)). Moment closure and parabolic/hyperbolic scaling techniques then yielded (see Supplementary Information for the full derivation):

(2)VC=χ01(K1+C)(K2+C)∇C

where *χ*_0_ is a chemotactic sensitivity coefficient as in the KS, LS and RTBL models and the oxygen concentrations *K*_1_ and *K*_2_ are traditionally interpreted as the dissociation constants for the receptor, which here is *HemAT.*^26^ Importantly, *K*_1_ and *K*_2_ represent the boundaries of the logarithmic sensing regime, because for oxygen concentrations such that *K*_1_≪*C*≪*K*_2_, equation (2) reduces to *V*_C_≈(*χ*_0_*/K*_2_)∇*C*/*C*. Therefore, our model predicts logarithmic sensing in the range of concentrations delimited by *K*_1_ and *K*_2_, but not outside this range, in line with our experimental observations.

The model’s ability to predict the observed bacterial distributions and the logarithmic sensing regime is not only qualitative, but also quantitative. We tested this by determining the concentrations *K*_1_ and *K*_2_ and the sensitivity *χ*_0_ by fitting the steady-state version of equation (1)—with *D*_B_ from experiments (Supplementary Figure S2) and *V*_C_ from equation (2)—to the entire data set of 33 steady-state bacterial distributions, *B*(*x*) (Figure 1b) (Materials and Methods). The best fit yielded *K*_1_=131 n mol/l, *K*_2_=196 μ mol/l and *χ*_0_=1.43×10^−3^ μm^2^/s. For these parameter values the model predicts *B*(*x*) accurately over the vast majority of the conditions tested, with an average error of 11% and only 1 out of 33 cases having an error >30%, and thus accurately captures the dependence of the CMC on oxygen conditions (Figure 3b, red dashed line). An empirical verification of the extent of the logarithmic sensing regime can be obtained by determining the range of experiments in which the CMC was within 10% of the maximum (Figure 3b, region between the dashed blue lines). Given the structural properties of the model (see discussion above), we expect *K*_1_ and *K*_2_ to approximate the two boundaries of the logarithmic sensing regime. Indeed, we observe an excellent agreement between the inferred dissociation constants (Figure 3b, solid blue lines) and the empirical estimates.

**Figure 4:**
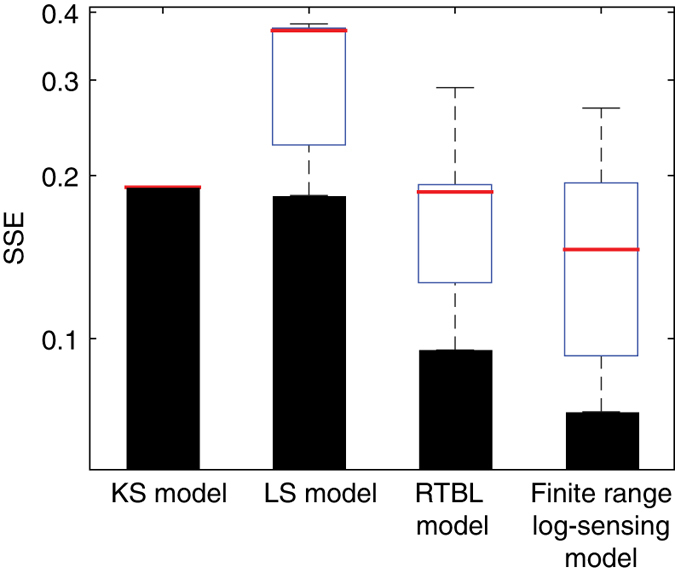
Error quantification in four classes of models for bacterial taxis, including the Keller–Segel model (KS), the Lapidus–Schiller model (LS), the Rivero–Tranquillo–Buettner–Lauffenburger model (RTBL), and the ‘finite range log-sensing’ model proposed here. For each model the graph shows the sum of the squared (weighted) errors (SSE, equation (5)) of the best solution identified among 100 iterations of the parameter optimization routine (black bars; Supplementary Information). The box plot summarizes the statistics of the 100 solutions obtained for each model: the median of the weighted SSE is in red, the extent of the blue box shows the first and last quartiles of the distribution, and the whiskers indicate the best and worst solution identified.

Repeating the fitting procedure to *B*(*x*) for the three classes of chemotaxis models proposed to date (KS, LS and RTBL; Supplementary Information, Supplementary Figures S3–S5) showed that the ‘finite range log-sensing’ model proposed here is the one that best predicts our observations (Supplementary Table S1), with a 23%–61% lower prediction error than the other models (Figure 4). Two remarks have to be made in this context: (i) models with a larger number of parameters will tend perform better than models with fewer parameters (see Figure 4: both the models with 2 parameters, namely RTBL and Finite range log-sensing, perform consistently better than KS and LS) and (ii) we note that it was only possible to distinguish between the performance of different models owing to the low levels of noise in the observations brought about by the very large number of bacterial positions recorded (Figure 1c). We speculate that the noise intrinsic in most data sets on microbial taxis to date has prevented such a comparative model identification, highlighting the potential of image analysis for the accurate characterization of microbial taxis behaviors. This quantitative grounding of the model then allows one to apply it to predict the steady-state distributions of bacteria over the entire spectrum of oxygen concentrations and (linear) gradients (Figure 3c).

**Figure 5:**
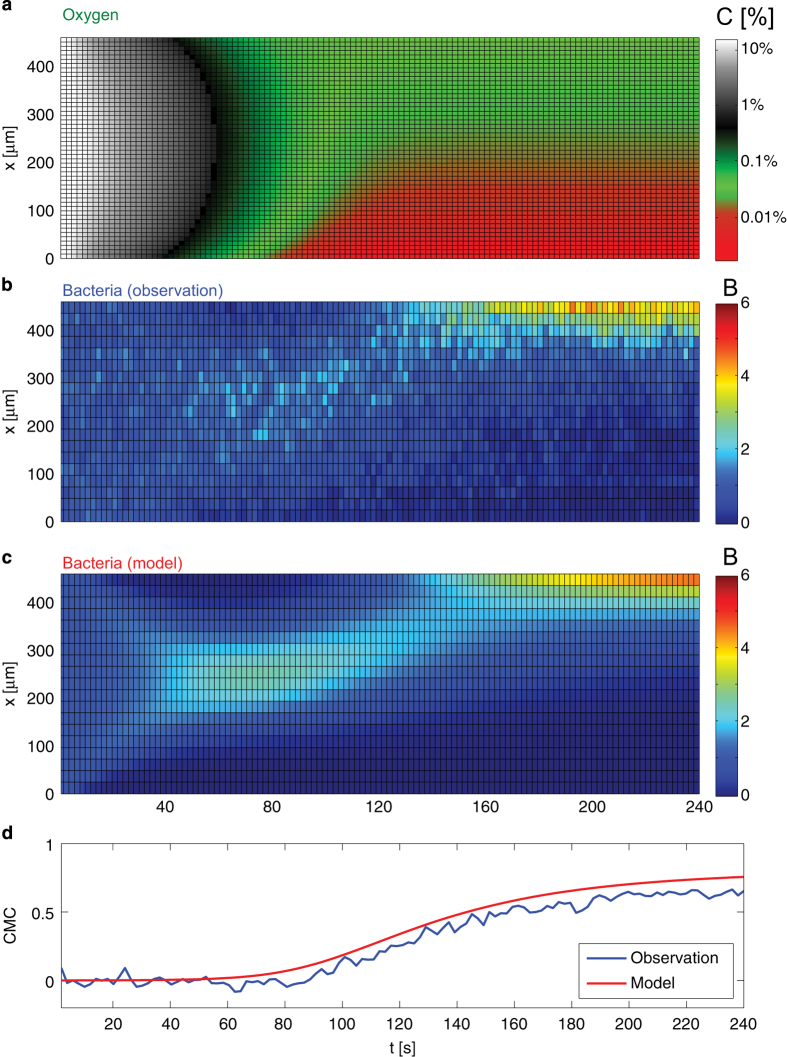
Validation of the ‘finite range log-sensing’ model on transient aerotaxis dynamics. (**a**) Simulated temporal evolution of the oxygen concentration across the test channel (*x*), as boundary conditions were changed from 21%–21% to 0%–0.05% oxygen saturation. (**b**) Observed and (**c**) predicted temporal evolution of the bacterial distribution across the test channel, *B*(*x*). (**d**) Temporal evolution of the Chemotactic Migration Coefficient (CMC) for the observed (blue) and predicted (red) transient aerotaxis response.

The most stringent validation of the model, which was derived based solely on steady-state data, was carried out by testing its performance in predicting the full aerotaxis dynamics in a transient experiment. In this experiment we exposed a *B. subtilis* population to a time-varying oxygen gradient beginning from uniform conditions (21%–21%) and ending after ~4 min with a gradient at low oxygen concentration (0%–0.05%) (Figure 5a). The spatial distribution of cells, *B*(*x*,*t*), was tracked over time (Materials and Methods) as the oxygen gradient formed, showing bacteria migrating towards the higher oxygen region (Figures 5b and d). The timing of the onset and completion of this migration, as well as the magnitude of the resulting accumulation (CMC), were successfully captured by the model, composed of Equations (1) and (2) (Figures 5c and d). Differences in the resolution of the band between experiments and model were mostly due to the impossibility of collecting the same large amounts of data in these transient experiments as in the steady-state experiments, owing to the temporal evolution of the bacterial concentration profile. The overall good agreement confirms the ability of the model to capture not only the steady-state aerotactic response of *B. subtilis*, but also the transient.

## Conclusions

We probed *B. subtilis*’ aerotactic response in a wide range of oxygen gradients and concentrations, with exquisite precision in the quantification of cell distributions permitted by collection of very large data sets. Our results robustly demonstrate that, in contrast to previous findings,^1^ *B. subtilis* always seeks the highest oxygen concentration available (Figure 2). Interestingly, we also observed that the strength of aerotaxis decreased above 20% oxygen—the maximum concentration of oxygen typically found in soil;^27^ this also being a potential indicator of a mechanism to limit exposure to reactive oxygen species and oxidative damage.

Short of only being capable of exploiting oxygen-rich environments, observations in the quasi-anoxic and micro-oxic range revealed that *B. subtilis* is capable of exploiting shallow gradients at low oxygen concentrations, where it still displays strong aerotaxis. This behavior may underpin the escape from anoxic conditions, such as those prevalent in the intraluminal area of warm blooded animal guts.^28^ This information is relevant in light of the recent identification of *B. subtilis* in mammalian microbiota.^29^

Our findings demonstrate that *B. subtilis* uses logarithmic sensing to measure and migrate in oxygen gradients and establishes the boundaries of the logarithmic sensing regime (Figures 2 and 3b). This property of chemoattractant signal transduction was previously found to underpin *E. coli*’s chemotaxis towards aspartate and serine.^23^ Our results then demonstrate that logarithmic sensing is present in species of bacteria beyond *E. coli* and is not limited to chemotaxis but can also apply to aerotaxis. A recent study in *C. crescentus*, based on single-cell trajectory analysis, also suggested that aerotaxis in that species obeys logarithmic sensing, yet this conclusion was based on a single oxygen gradient.^6^ Our comparison of different mathematical models suggests that such inferences of logarithmic sensing must be made with caution. Data over a broad range of oxygen conditions and a large number of cells are necessary to reliably distinguish between different models, and only some models support logarithmic sensing. Furthermore, the data-intensive approach presented here also provides information on the oxygen regime where logarithmic sensing applies, which is important in determining the role and occurrence of this rescaling behavior in natural environments.

Logarithmic sensing is particularly advantageous in the diffusion-dominated microscale world, where information about the position of an oxygen source is encoded in the shape of the oxygen field rather than in its absolute magnitude.^30^ Furthermore, a wide range of oxygen concentrations and gradients are likely to characterize the natural habitats of *B. subtilis,* making logarithmic sensing an effective feature of a cosmopolitan lifestyle. We speculate that, by greatly improving the effectiveness of the bacterial search for essential compounds such as oxygen, logarithmic sensing in aerotaxis may be an important factor in the success and abundance of species such as *B. subtilis*.

## Materials and methods

### Design and execution of the experiments

*B. subtilis* strain OI1085 was obtained from George Ordal’s laboratory. Cells from a frozen (−80 °C) stock were resuspended in 2 ml of Cap Assay Minimal media,^31^ grown in a shaking incubator at 37 °C, 250 r.p.m. until the culture reached OD_600_=0.3. The bacterial suspension was then diluted 1:10 in fresh media before the injection into the microfluidic device (note that there were no modifications to the ambient air available to the cell cultures, see Methods in the Supplementary Information, Supplementary Figure S10 and the Supplementary Video). Cells were then exposed to the desired oxygen gradient by flowing mixtures of oxygen/nitrogen in the source and sink channels (Figures 1 and 5). For steady-state measurements of *B(x)* (Figure 1) cells were allowed to explore the gradient for 5 min and then 30 000 phase contrast micrographs were taken at intervals of 67 ms (such settings were empirically identified to provide the best compromise between a reasonable duration of each experiment, the accuracy in the estimate of *B*(*x*) and size of the resulting data set). To quantify the transient aerotactic response of *B. subtilis* (Figure 5) we injected cells in the test channel (21% oxygen flowing in the sink and source channels) and applied a 0% O_2_ to the sink and 0.05% O_2_ in the source. A video was then acquired at 100 frames per second for 4 min to locate cells during their migration in response to the developing oxygen gradient.

### Data analysis and model identification

Single-cell coordinates were obtained from micrographs as previously described.^32^ In the steady-state experiments all the coordinates from the 30 000 images acquired were pooled together to obtain the profiles plotted in Figure 1 (Supplementary Materials and Methods). For the model validation experiment, instead, the coordinates from 200 consecutive frames (acquired over 2 s) were pooled together to calculate a single *B(x)*, see Figure 5. The optimal parameters of the mathematical models included in this comparison were obtained via a fitting procedure accomplished using a Genetic Algorithm implemented in MATLAB. This routine was designed to explore the parameter space and identify the combination of parameter values that minimized the discrepancy, calculated as the weighted Sum of the Squared Error (SSE), between predicted and measured *B(x)* in response to a specific gradient profile (Supplementary Materials and Methods).
