# Supplementary material for: Logarithmic sensing in Bacillus subtilis aerotaxis
Source: NPJ Syst Biol Appl. 2017 Jan 19;3:16036–. doi: 10.1038/npjsba.2016.36 (PMC5516866; doi:10.1038/npjsba.2016.36)
Supplement: Supplementary Information [file npjsba201636-s1.pdf]

# Supplementary Information

Filippo Menolascina[1,2], Roberto Rusconi[3,4], Vicente I. Fernandez[3,4], Steve P. Smriga[3,4], Zahra Aminzare[5], Eduardo D. Sontag[6] & Roman Stocker[3,4]

[1]*Institute for Bioengineering, School of Engineering, The University of Edinburgh, EH9 3DW Edinburgh, Scotland, UK*

[2]*SynthSys - Centre for Synthetic and Systems Biology, The University of Edinburgh, EH9 3BF Edinburgh, Scotland, UK*

[3]*Institute of Environmental Engineering, Department of Civil, Environmental and Geomatic Engineering, ETH Zurich, 8093 Zurich, Switzerland*

[4]*Ralph M. Parsons Laboratory, Department of Civil and Environmental Engineering, Massachusetts Institute of Technology, Cambridge, MA 02139, USA*

[5]*The Program in Applied and Computational Mathematic, Fine Hall, Washington Road, Princeton, NJ 08544, USA*

[6]*Department of Mathematics, Hill Center, 110 Frelinghuysen Rd, Rutgers, The State University of New Jersey, Piscataway, NJ 08854, USA*

## SI Results

**Oxgen diffusion within the device** Oxygen diffusion within the microfluidic device was studied combining in-silico simulations and in-vitro experiments. To this aim a 1D model was developed

in COMSOL Multiphysics 4.4 (see Materials and Methods). Oxygen diffusion dynamics in the test channel were simulated for two gradients: 0%-20% and 0%-10% oxygen (dashed lines in Fig. S1). We then set out to quantify how the spatial profile of oxygen varied as a function of time for both gradients.

To measure oxygen concentrations in the test channel we flowed in the test channel a 167 ppm solution of ruthenium tris(2,2'-dypiridyl) dichloride hexahydrate (RTDP) in 66% ethanol in water, at a flow rate of 200 nL/min. RTDP is a fluorescent dye sensitive to oxygen: the larger the oxygen concentration, the smaller the intensity of the fluorescence that RTDP emits. Consistently with previous studies[16] we used the Stern-Volmer equation $I_0/I = 1 + K_q[O_2]$ to convert the fluorescence intensity $I$ in an oxygen concentration $[O_2]$. First we need to estimate $I_0$, the fluorescence intensity in absence of oxygen (100% nitrogen) and the quenching constant $K_q$. To do so we flowed pure nitrogen (0% oxygen) in the source and sink channels, waited 10 minutes to make sure the gas concentration in the channel was equilibrated to uniform, and then acquired a fluorescence image of the channel. Background estimation and correction was carried out as in[16]; to this aim we extracted background fluorescence in the test channel fitting a second order polynomial across the x axis (i.e. the direction of the gradient) to intensities of areas 100 $\mu$m in the left and right PDMS walls -as there is no dye in the PDMS, and PDMS is not autofluorescent at the RTDP emission wavelength, we reasoned that any fluorescence in these areas can be classified as background. This procedure yielded an estimate of background fluorescence in the test channel -obtained using the fitted polynomial- that we used for correction by subtraction to all the intensity profiles we acquired[16]. As commonly noted, the quality of the micrographs decreased quickly in the vicinity

of PDMS walls; as this made a reliable measurements of signals very close to the boundaries of the test channel challenging, we decided to analyse oxygen concentrations between 10 and 450 $\mu$m. In the same manner we measured a second reference intensity, $I_{air}$, by flowing air (20.8% oxygen) in both the source and sink channels. This allowed us to calculate the quenching constant $K_q$ by inverting the Stern-Volmer equation and plugging in the measurements of $I_0$ and $I = I_{air}$. This yielded$K_q = (I_0/I_{air} - 1)/20.8\% = 6.02$. With this value of $K_q$, any generic value of RTDP intensity $I$ can be converted in an oxygen concentration solving the Stern-Volmer equation for the oxygen concentration, $[\mathrm{O}_2] = (I_0/I - 1)/K_q$.

To assess the accuracy of our mathematical model in predicting the spatiotemporal profile of oxygen, we generated (in two separate experiments) the two gradients simulated with our model, namely 0%-10% and 0%-20% oxygen. For each case, we quantified the background fluorescence, flowed in the source and sink the gas mixes appropriate to generate the desired gradient (e.g. nitrogen in the sink and 20% oxygen in the source for 0%-20%) and acquired fluorescence images every 10 seconds for 5 minutes. We then converted the fluorescence intensity values into oxygen concentrations with the procedure described above. The results of this approach are presented in Fig. S1 (solid line in panel A and squares in panel B). These measurements confirm that (i) the steady-state oxygen profile in the device is indeed linear, and (ii) both the steady-state (Fig. S1A) and the transients of oxygen diffusion (Fig. S1B) are well predicted by the mathematical model.

We note that, in the device used for our experiments, if we denote by $O_{\mathrm{source}}$ and $O_{\mathrm{sink}}$ the concentration (expressed in %) of oxygen flown in the source and sink channels, respectively, cells are exposed to >90% of the gradient from $O_{\mathrm{sink}}$ to $O_{\mathrm{source}}$, and <10% of the gradient occurs within

the lateral PDMS boundaries separating the source and sink channels from the test channel. This can be easily observed in Table 1. When $O_{\mathrm{source}}$ =100% and $O_{\mathrm{sink}}$ =0%, the boundary conditions in the test channel are C(0 $\mu$m)= 6.04E-5 M, i.e. 4.7% of 1.3E-3 M (oxygen saturation in water in the lab), and C(460 $\mu$m)= 1.24E-3 M, i.e. 95.4% of 1.3E-3 M. This corresponds to a total drop in oxygen concentration within the test channel of ∼90.7%, to be compared to a 100% drop between the source and sink channels. This also means that $\sim 9.3\%$ of the gradient is retained in the PDMS walls and is not available to the cells.

**Bacterial diffusivity** $D_B$ In order to measure the diffusivity of *B. subtilis* we tracked and analyzed bacterial trajectories in uniform concentrations of oxygen ranging from 0% to 100% (Fig. S2).The (2D projection) Mean Squared Displacement (MSD) of cell, subjected rotational can be written as:

$$\mathrm{MSD}(t) = \frac{V^2 \tau_R^2}{2}\left(\frac{2t}{\tau_R} + e^{-2t/\tau_R} - 1\right) \tag{1}$$

where $V$ is cell's swimming speed, $t$ is time and $\tau_R$ is the characteristic time-scale associated to rotational diffusion. We measure $V$ directly (Fig. S2C) from bacterial trajectories and obtain $\tau_t$ and, therefore $\tau_R$ via fitting [33 ,32]. In agreement with what has been reported in literature [34] we measure a tumbling time $\tau_t = \frac{1}{2}\tau_R \simeq 0.71s$ at low oxygen concentrations ($O_2$ <1%) and higher tumbling times $\tau_t \simeq 1.18s$ for $O_2 > 1\%$ (Fig. S2B). Consistently with previous reports our data also suggest the swimming speed increases with the concentration of oxygen (see Fig. S2C) up to ∼1% $O_2$. We can use these observations to derive the translational diffusion coefficient:

$$D_B = \frac{V^2\, \tau_t}{2} \tag{2}$$

We found that the translational diffusion coefficient shows a roughly constant value (336 $\mu$m$^2$/s) between 30% and 100% $O_2$. An additional constant $D_B$ regime can be identified at lower $O_2$ concentration $D_B \simeq 181$ $\mu$m$^2$/s for 0%$<$ $O_2$ $\leq$1%, while at intermediate $O_2$ concentrations (1%$<$ $O_2$ $<$30%) $D_B$ rapidly increases and decreases.

**Mechanistic derivation of advection-diffusion equation** In this section, we will show how an advection-diffusion equation for densities, of the type that we fit to data, might be reasonable. As little is known about the mechanistic basis of *B. subtilis* aerotaxis [35] our approach is as follows. We will first review an accepted and experimentally validated model of *E. coli*, and show how it leads to an advection-diffusion equation of the desired form. We will then see how this mechanism would be modified by incorporating knowledge about the differences between *E. coli* and *B. subtilis* chemotaxis, and we will show that the same advection-diffusion equation results in spite of this difference (albeit with very different parameters). As aerotaxis and chemotaxis in *B. subtilis* employs the same receptor mechanism [11], we will postulate that this same model applies to aerotaxis.

We organize this section by first discussing a general approach to advection-diffusion approximations, before specializing to the *E. coli* and *B. subtilis* models.

**Preliminaries** Let $p(x, y, \nu, t)$ be a density function describing a population of "particles" or agents (for example, bacteria), modeled in a $2N + m$ dimensional phase space, where at time $t$, $x = (x_1, \ldots, x_N) \in \mathbb{R}^N$ ($N = 1, 2, 3$; we soon specialize to $N = 1$) denotes the position of the agent, $y = (y_1, \ldots, y_m) \in Y \subset \mathbb{R}^m_{\geq 0}$ denotes the internal states of the agent (we will soon specialize to $m = 1$), and $\nu \in V \subset \mathbb{R}^N$ denotes its velocity. Also, $S(x) = (S_1, \ldots, S_M) \in \mathbb{R}^M$ denotes the concentration of signals in the environment which are sensed by each agent at space location $x$ (we will soon specialize to $M = 1$). The external signal $S$ is assumed to be constant in time (steady state assumption on chemoattractant), but is allowed to depend on space coordinates.

We assume that the following system of ordinary differential equations describes the evolution of the intracellular state, in the presence of the extracellular signal $S(x)$ at the current location of the agent:

$$\frac{dy}{dt} = f(y, \nu, S(x), S'(x)), \tag{3}$$

where $f \colon \mathbb{R}^m \times \mathbb{R}^N \times \mathbb{R}^M \times \mathbb{R}^M \to \mathbb{R}^m$ is a continuously differentiable function with respect to each component, i.e., $f \in C^1(\mathbb{R}^m \times \mathbb{R}^N \times \mathbb{R}^M \times \mathbb{R}^M)$. The derivative $S'(x)$ indicates derivative of $S$ with respect to space (local gradient of chemoattractant). In most models, $f$ depends explicitly only on $y$ and $S$, but we allow this additional generality in the theory.

We assume also given an instantaneous reorientation ("tumbling") rate $\lambda = \lambda(y, S(x), S'(x))$ (often, $\lambda$ depends only on certain combinations of $y$ and $S(x)$, represented by the "activity" of re-

ceptors), the evolution of $p$ is governed by the following transport (or "Fokker-Planck" or "forward Kolmogorov") equation [36] (omitting arguments of functions $p$ and $f$, for readability):

$$\frac{\partial p}{\partial t} + \nabla_x \cdot \nu p + \nabla_y \cdot f p = -\lambda(y, S(x), S'(x))p + \int_V \lambda(y, S(x), S'(x)) T(y, \nu, \nu') p(x, y, \nu', t)\, d\nu' \quad (4)$$

where the nonnegative kernel $T(y, \nu, \nu')$ is the probability that the agent changes the velocity from $\nu'$ to $\nu$ if a change of direction occurs. Also $\int_V T(y, \nu, \nu')\, d\nu = 1$.

The main goal here is to derive an approximate macroscopic model for chemotaxis using the microscopic model (4), i.e., we want to find an equation to approximately describe the evolution of the marginal density:

$$n(x, t) \;=\; \int_V \int_Y p(x, y, \nu, t)\, dy d\nu, \quad (5)$$

by adapting methods from Grunbaum [24] and Othmer [25]. We will assume that the external signal is isotropic in two state directions, so that in effect we can study one-dimensional motion.

**A general equation in one dimension** From now on, we study the movement of agents in one dimension have constant speed, so that the velocities are $\nu \in \{\bar{\nu}, -\bar{\nu}\}$, where $\bar{\nu}$ is a positive number, which we'll think of as a parameter in the equations. We will write $f^+(y, \bar{\nu}, S, S')$ instead of $f(y, \bar{\nu}, S, S')$ and $f^-(y, \bar{\nu}, S, S')$ instead of $f(y, -\bar{\nu}, S, S')$, and omit the bars from $\bar{\nu}$ from now

on. Similarly, for $p$, we let $p^{\pm}(x, y, t)$ denote the density of particles that at time $t$, are located at position $x$, with the internal state $y$, and with the constant speed $\nu$, and moving to the right (+) or left (−) respectively.

The internal state evolves according to the following ODE system:

$$\frac{dy}{dt} = f^{\pm}(y, \nu, S, S'), \tag{6}$$

where $f^{\pm}\colon \mathbb{R}_{\geq 0} \times \mathbb{R} \times \mathbb{R} \times \mathbb{R} \to \mathbb{R}$ are continuously differentiable functions in each argument that describe the evolution of internal state of agents which move to the right (+) and left (−) respectively.

Note that we are allowing $f$ to depend on the direction of movement as well as $\nu$ and $S'$, the derivative of $S$ with respect to space. In our examples, $f^+ = f^-$ only depends on $y$ and $S$, but we can consider the more general dependence in these preliminary derivations.

We describe the tumbling rate by introducing:

$$\lambda(y, S, S') = g(y, S, S'), \tag{7}$$

where $g$ is a continuous function.

Then, according to Equation (4), $p^{\pm}(x, y, t)$ satisfy the following coupled first-order partial differ-

ential equations:

$$\frac{\partial p^+}{\partial t} + \nu \frac{\partial p^+}{\partial x} \;+\; \frac{\partial}{\partial y}\left[f^+(y,\nu,S,S')\,p^+\right] \;=\; g(y,S,S')(-p^+ + p^-) \tag{8}$$

$$\frac{\partial p^-}{\partial t} - \nu \frac{\partial p^-}{\partial x} \;+\; \frac{\partial}{\partial y}\left[f^-(y,\nu,S,S')\,p^-\right] \;=\; g(y,S,S')(p^+ - p^-). \tag{9}$$

See [25] for existence and uniqueness of solutions of (8)-(9)

We assume given a forward-invariant set $I \subset \mathbb{R}_{\geq 0}$, i.e., if $y(0) \in I$, then $y(t) \in I$, for all $t \geq 0$, with the property that $p_0^{\pm}(x,y)$ are supported on $I$, i.e., $p_0^{\pm}(x,y) = 0$, when $y \notin I$. (In each of the examples to be considered below, such a set $I$ will be constructed, by appealing to Lemma 1 in Section  below). In other words,

$$p^{\pm}(x,y,t) = 0, \quad \forall x, y \notin I, t \geq 0. \tag{10}$$

The objective is to derive an approximate equation for the macroscopic density function

$$n(x,t) \;=\; \int_{\mathbb{R}_{\geq 0}} p^+(x,y,t) + p^-(x,y,t)\;dy, \tag{11}$$

using the microscopic model (8)-(9), by adapting a technique from [25]. To this end we introduce a flux variable $j$ as well as moments associated to $n$ and $j$:

$$
\begin{aligned}
j(x,t) &= \int_{\mathbb{R}\geq 0} \nu \left(p^+(x,y,t) - p^-(x,y,t)\right)\, dy, \\
n_i(x,t) &= \int_{\mathbb{R}\geq 0} y^i \left(p^+(x,y,t) + p^-(x,y,t)\right)\, dy, \quad \text{for} \quad i = 1,2,\ldots \\
j_i(x,t) &= \int_{\mathbb{R}\geq 0} y^i \nu \left(p^+(x,y,t) - p^-(x,y,t)\right)\, dy, \quad \text{for} \quad i = 1,2,\ldots.
\end{aligned} \tag{12}
$$

Note that by Equation (10) all the moments are well defined.

Next, we assume $f^+ = f_0 + \nu f_1$, and $f^- = f_0 - \nu f_1$, where the Taylor expansions of $f_0$ and $f_1$, with respect to the internal state $y$, are given as follows:

$$
f_0 = A_0 + A_1 y + A_2 y^2 + \cdots, \tag{13}
$$

$$
f_1 = B_0 + B_1 y + B_2 y^2 + \cdots, \tag{14}
$$

for some $A_i$'s and $B_i$'s that are functions of $S$, $S'$, and $\nu^2$. (We formally assume that these expansions exist.) Also we consider the following Taylor expansion for $g(y,S,S')$:

$$
g(y,S,S') = a_0 + a_1 y + a_2 y^2 + \cdots, \tag{15}
$$

where the $a_i$'s are functions of $S$, and $S'$.

In addition, we assume $A_0 = 0$, because this is satisfied in our examples. Then by multiplying

(8) and (9) by 1, $\nu$, and/or $y$, adding or subtracting, and integrating with respect to $y$ on $\mathbb{R}_{\geq 0}$, and applying the fundamental theorem of calculus and integration by parts, we obtain the following equations for macroscopic density and flux and their first moments:

$$\begin{aligned}
\frac{\partial n}{\partial t}+\frac{\partial j}{\partial x} &= 0, && (16)\\
\frac{\partial j}{\partial t}+\nu^2\frac{\partial n}{\partial x} &= -2a_0 j-2a_1 j_1-2\sum_{k\geq 2}a_k j_k, && (17)\\
\frac{\partial n_1}{\partial t}+\frac{\partial j_1}{\partial x} &= B_0 j+A_1 n_1+B_1 j_1+\sum_{k\geq 2}A_k n_k+\sum_{k\geq 2}B_k j_k, && (18)\\
\frac{\partial j_1}{\partial t}+\nu^2\frac{\partial n_1}{\partial x} &= \nu^2 B_0 n+\nu^2 B_1 n_1+(A_1-2a_0)j_1 && (19)\\
&\quad +\nu^2\sum_{k\geq 2}B_k n_k+\sum_{k\geq 2}(A_k-2a_{k-1})j_k
\end{aligned}$$

Note that by Equation (10), $p^{\pm}=0$ outside the interval $I$, therefore, for any $i=0,1,\ldots$

$$\lim_{y\to\infty}y^i(p^+\pm p^-) \;=\; 0, \quad \lim_{y\to 0}y^i(p^+\pm p^-) \;=\; 0.$$

**Parabolic scaling**

In this section, we introduce a parabolic scaling to derive an approximate chemotaxis equation from the moment equations (16)-(19). Let $L$, $T$, $\nu_0$, $y_0$, and $N_0$ be scale factors for the length, time, velocity, internal state, and particle density respectively, and define the following dimensionless

parameters (we use hats to denote the dimensionless forms of the parameters):

$$\hat{\nu} = \frac{\nu}{\nu_0}, \quad \hat{y} = \frac{y}{y_0}, \tag{20}$$

$$\hat{n} = \frac{n}{y_0 N_0}, \quad \hat{j} = \frac{j}{y_0 N_0 \nu_0}, \tag{21}$$

$$\hat{n}_i = \frac{n_i}{y_0^{i+1} N_0}, \quad \hat{j}_i = \frac{j_i}{y_0^{i+1} N_0 \nu_0}, \quad \text{for} \quad i = 1, 2, \dots \tag{22}$$

$$\hat{a}_i = y_0^i T\, a_i, \quad \hat{A}_i = y_0^{i-1} T\, A_i, \quad \hat{B}_i = y_0^{i-1} L\, B_i, \quad \text{for} \quad i = 0, 1, \dots \tag{23}$$

The parabolic scales of space and time are given by:

$$\hat{x} = \left(\frac{\epsilon L}{\nu_0 T}\right) \frac{x}{L}, \quad \hat{t} = \epsilon^2 \frac{t}{T}, \tag{24}$$

for any arbitrary $\epsilon$.

Now assume that under appropriate conditions to be verified in particular examples, for any $i \geq 2$, the $j_i$'s and $n_i$'s are much smaller than $j_1$ and $n_1$ and can be neglected. (For example see the definition of shallow gradient in Example  below.)

Therefore, the dimensionless form of moment equations (16)-(19), for $\epsilon = \dfrac{T\nu_0}{L}$, become:

$$\epsilon^2 \frac{\partial \hat{n}}{\partial \hat{t}} + \epsilon \frac{\partial \hat{j}}{\partial \hat{x}} \ = \ 0, \tag{25}$$

$$\epsilon^2 \frac{\partial \hat{j}}{\partial \hat{t}} + \epsilon \hat{\nu}^2 \frac{\partial \hat{n}}{\partial \hat{x}} \ = \ -2\hat{a}_0 \hat{j} - 2\hat{a}_1 \hat{j}_1, \tag{26}$$

$$\epsilon^2 \frac{\partial \hat{n}_1}{\partial \hat{t}} + \epsilon \frac{\partial \hat{j}_1}{\partial \hat{x}} \ = \ \epsilon \hat{B}_0 \hat{j} + \hat{A}_1 \hat{n}_1 + \epsilon \hat{B}_1 \hat{j}_1, \tag{27}$$

$$\epsilon^2 \frac{\partial \hat{j}_1}{\partial \hat{t}} + \epsilon \hat{\nu}^2 \frac{\partial \hat{n}_1}{\partial \hat{x}} \ = \ \epsilon \hat{\nu}^2 \hat{B}_0 \hat{n} + \epsilon \hat{\nu}^2 \hat{B}_1 \hat{n}_1 + (\hat{A}_1 - 2\hat{a}_0)\hat{j}_1. \tag{28}$$

Next, we write Equations (25)-(28) in a matrix form, as follows:

$$\epsilon^2 \frac{\partial \hat{w}}{\partial \hat{t}} + \epsilon \frac{\partial}{\partial \hat{x}} P \hat{w} \ = \ \epsilon Q \hat{w} + R \hat{w}, \tag{29}$$

where $\hat{w} \ = \ \left(\hat{n}, \hat{j}, \hat{n}_1, \hat{j}_1\right)^T$ and the matrices $P$, $Q$, and $R$ defined as follows:

$$P = \begin{pmatrix} 0 & 1 & 0 & 0 \\ \hat{\nu}^2 & 0 & 0 & 0 \\ 0 & 0 & 0 & 1 \\ 0 & 0 & \hat{\nu}^2 & 0 \end{pmatrix}, Q = \begin{pmatrix} 0 & 0 & 0 & 0 \\ 0 & 0 & 0 & 0 \\ 0 & \hat{B}_0 & 0 & \hat{B}_1 \\ \hat{\nu}^2 \hat{B}_0 & 0 & \hat{\nu}^2 \hat{B}_1 & 0 \end{pmatrix}, R = \begin{pmatrix} 0 & 0 & 0 & 0 \\ 0 & -2\hat{a}_0 & 0 & -2\hat{a}_1 \\ 0 & 0 & \hat{A}_1 & 0 \\ 0 & 0 & 0 & \hat{A}_1 - 2\hat{a}_0 \end{pmatrix}.$$

Assuming the regular perturbation expansion for $w$,

$$\hat{w} \ = \ \hat{w}^0 + \epsilon \hat{w}^1 + \epsilon^2 \hat{w}^2 + \ldots, \quad \text{where} \quad \hat{w}^i \ = \ \left(\hat{n}^i, \hat{j}^i, \hat{n}^i_1, \hat{j}^i_1\right)^T,$$

and comparing the terms of equal order in $\epsilon$ in (29), we get:

$$\epsilon^0 : \quad R\hat{w}^0 \;=\; 0 \quad \Rightarrow \quad \hat{w}^0 \;=\; (\hat{n}^0, 0, 0, 0)^T \tag{30}$$

$$\epsilon^1 : \quad R\hat{w}^1 \;=\; -Q\hat{w}^0 + \frac{\partial}{\partial \hat{x}} P\hat{w}^0$$

$$\Rightarrow \quad \begin{pmatrix} 0 \\ \\ -2\hat{a}_0\hat{j}^1 - 2\hat{a}_1\hat{j}_1^1 \\ \\ \hat{A}_1\hat{n}_1^1 \\ \\ (\hat{A}_1 - 2\hat{a}_0)\hat{j}_1^1 + \hat{\nu}^2\hat{B}_0\hat{n}^0 \end{pmatrix} = \begin{pmatrix} 0 \\ \\ \hat{\nu}^2\frac{\partial}{\partial \hat{x}}\hat{n}^0 \\ \\ 0 \\ \\ 0 \end{pmatrix}. \tag{31}$$

From the last equality of Equation (31), we can derive the following equation for $\hat{j}_1^1$:

$$\hat{j}_1^1 \;=\; -\frac{\hat{\nu}^2\hat{B}_0}{\hat{A}_1 - 2\hat{a}_0}\hat{n}^0.$$

By substituting $\hat{j}_1^1$ into the second equality of Equation (31), we obtain the following equation

$$\hat{j}^1 \;=\; -\frac{\hat{\nu}^2}{2\hat{a}_0}\frac{\partial \hat{n}^0}{\partial \hat{x}} + \frac{\hat{a}_1\hat{B}_0\hat{\nu}^2}{\hat{a}_0(\hat{A}_1 - 2\hat{a}_0)}\hat{n}^0. \tag{32}$$

Now we compare the terms with order $\epsilon^2$:

$$\epsilon^2 : \quad R\hat{w}^2 \;=\; -Q\hat{w}^1 + \frac{\partial}{\partial \hat{x}}P\hat{w}^1 + \frac{\partial}{\partial \hat{t}}\hat{w}^0. \tag{33}$$

Note that $(1, 0, 0, 0)^T$ is in the kernel of $R$ and the right hand side of (33) is in the image of $R$.

Therefore their inner product is zero:

$$\frac{\partial}{\partial \hat{x}} \hat{j}^1 + \frac{\partial}{\partial \hat{t}} \hat{n}^0 \ = \ 0. \tag{34}$$

Equation (32) together with Equation (34) give the following equation for $n^0$ in the dimensionless variables:

$$\frac{\partial \hat{n}^0}{\partial \hat{t}} \ = \ \frac{\partial}{\partial \hat{x}} \left( \frac{\hat{\nu}^2}{2\hat{a}_0} \frac{\partial \hat{n}^0}{\partial \hat{x}} - \frac{\hat{a}_1 \hat{B}_0 \hat{\nu}^2}{\hat{a}_0(\hat{A}_1 - 2\hat{a}_0)} \hat{n}^0 \right). \tag{35}$$

Since $n(x,t) = n^0(x,t) + \mathcal{O}(\epsilon)$, if we neglect the $\mathcal{O}(\epsilon)$ term, Equation (35) leads to the following chemotaxis equation in dimensionless variables:

$$\frac{\partial \hat{n}}{\partial \hat{t}} \ = \ \frac{\partial}{\partial \hat{x}} \left( \frac{\hat{\nu}^2}{2\hat{a}_0} \frac{\partial \hat{n}}{\partial \hat{x}} - \frac{\hat{a}_1 \hat{B}_0 \hat{\nu}^2}{\hat{a}_0(\hat{A}_1 - 2\hat{a}_0)} \hat{n} \right). \tag{36}$$

Changing back to the original (dimensional) variables, we obtain the following PDE:

$$\frac{\partial n}{\partial t} \ = \ \frac{\partial}{\partial x} \left( \frac{\nu^2}{2a_0} \frac{\partial n}{\partial x} - \frac{a_1 B_0 \nu^2}{a_0(A_1 - 2a_0)} n \right). \tag{37}$$

**Examples**

***E.coli***

The following simplified one-dimensional model provides a phenomenologically accurate model of the chemotactic response of *E.coli* bacteria to MeAsp; see for example [39], [37]. The internal state evolves according to an ordinary differential equation:

$$\frac{dm}{dt} = K_r(1-a) - K_b a$$

which describes the methylation state of receptors, where $a$ is a number between 0 and 1 that quantifies the fraction of active receptors, and is written as follows:

$$a = \frac{1}{1 + (F_m F_l)^N}$$

in terms of free energy differences due to methylation and ligand respectively:

$$F_m = \exp(\alpha(1-m)) \,, \qquad F_l = \frac{1 + S/K_I}{1 + S/K_A} \,,$$

where $K_I$ and $K_A$ are dissociation constants for inactive and active Tar receptors, respectively. This arises from an MWC [38] model of clusters of $N$ receptors that rapidly switch between active and inactive states, In summary, we write:

$$a = \frac{1}{1 + K \left( \dfrac{S + K_I}{(S + K_A)\, y} \right)^N}$$

and $K$, $K_I$, and $K_A$ are nonnegative constants and $K_I < K_A$.

With appropriate parameter choices [39], [37], this model fits very well the response of *E. coli* to the ligand $\alpha$-methylaspartate.

*E. coli* tumbling rate is controlled by the concentration of cheY-P. In this simplified model, one thinks of phosphorylation state of cheY as directly proportional to activity, assuming fast phosphotransfer. Thus, one takes the jump (or "tumbling" for bacteria) rate in the form:

$$\lambda(y, S) \;=\; \frac{1}{\tau} \left( \frac{a}{a_0} \right)^H .$$

Here $a_0$ denotes a steady-state kinase activity, $H$ a motor amplification coefficient, and $\tau$ the average run time. We write

$$\lambda(y, S) \;=\; R a^H, \tag{38}$$

where $R = (\tau a_0^H)^{-1}$.

It is convenient to use $y = e^{\alpha m}$ as a state variable, instead of the methylation level $m$. So the

equations can be rewritten as follows:

$$\frac{dy}{dt} \;=\; \alpha y \left( K_r(1-a) - K_b a \right) \;=\; py(q-a), \tag{39}$$

provided that we pick

$$p = \alpha(K_r + K_b)\,, \quad q = \frac{K_r}{K_r + K_b}\,.$$

Observe that $F_m = e^{\alpha}/y$ when expressed in terms of the new variable $y$. The parameters $p$, $q$, $K$, $N$, and $H$ are all positive, and, from its definition, it is clear that $q$ is between 0 and 1.

The objective is to derive a parabolic equation for the macroscopic density function. It is convenient to define a new internal state variable as follows:

$$w \;=\; p(a-q). \tag{40}$$

Then, a simple calculation shows that

$$\frac{dw}{dt} \;=\; \frac{N}{p}(w+pq)(w+pq-p)\left( w \pm \frac{\nu S'\left(K_A - K_I\right)}{\left(K_A + S\right)\left(K_I + S\right)} \right), \tag{41}$$

and

$$\lambda(w) \;=\; \frac{R}{p^H}(w+pq)^H. \qquad (42)$$

For convenience of notation, let us define $G(S) := \log\left(\dfrac{S+K_I}{S+K_A}\right)$.

**Lemma 1.** *Let* $c = \min\{pq, p-pq\}$. *If* $|G'(S)| \leq \frac{c}{\nu}$ *and* $|w(0)| \leq c$, *then* $|w(t)| \leq c$ *for all* $t \geq 0$.

See [57] for a proof.

Let $L$, $T$, $\nu_0$, and $N_0$ be scale factors for the length, time, velocity, and particle density respectively, and define the following dimensionless quantities: A simple calculation shows that:

$$\begin{aligned}
&G'(S)\widehat{G'(S)} \;=\; LG'(S), \quad \hat{N} \;=\; N, \quad \hat{p} \;=\; Tp, \quad \hat{w} = Tw, \quad \hat{q} \;=\; q \\
&\hat{R} \;=\; TR, \quad \hat{K}_A = \frac{K_A}{L}, \quad \hat{K}_I = \frac{K_I}{L}, \quad \hat{z} = Tz.
\end{aligned} \qquad (43)$$

All other parameters remain the same as in Equations (20)-(22), and Equation (24), for $y_0 = \frac{1}{T}$.

Note that for $\epsilon = \dfrac{\nu_0 T}{L}$, we have the following analogous result to Lemma 1, in hyperbolic scale:

$$\left|G'(S)\widehat{G'(S)}\right| \;\leq\; \frac{\hat{c}}{\hat{\nu}}\frac{1}{\epsilon}, \quad \hat{w}(0) \leq \hat{c} \quad \Rightarrow \quad \hat{w}(t) \leq \hat{c}, \;\; t > 0. \qquad (44)$$

**Definition 1** (shallow condition)**.** *If* $\left|G'(S)\widehat{G'(S)}\right| \;\leq\; \bar{K}$, *where* $\bar{K} \;=\; \mathcal{O}(1)$, *we say* $S$ *has a shallow gradient.*

**Lemma 2.** *Assume that*

$$\left|G'(S)\widehat{G'(S)}\right| \leq \frac{\hat{c}}{\hat{\nu}}, \tag{45}$$

*i.e., $S$ has a shallow gradient. Then, for any $i \geq 1$,*

$$\frac{\hat{j}_i}{\hat{n}} \leq \mathcal{C}_i \epsilon^i, \quad \textit{and} \quad \frac{\hat{n}_i}{\hat{n}} \leq \mathcal{D}_i \epsilon^i,$$

*for some constants $\mathcal{C}_i = \mathcal{O}(1)$, and $\mathcal{D}_i = \mathcal{O}(1)$.*

See [57] for a proof.

**Remark 1.** Equation (45) is equivalent to the following condition for $G'(S)$:

$$|G'(S)| \leq \frac{c}{\nu}\epsilon, \tag{46}$$

or equivalently

$$\frac{\nu}{c}\left|\frac{(K_A - K_I)\,S'}{(S+K_A)\,(S+K_I)}\right| \leq \epsilon. \tag{47}$$

Note that for exponential signal $S(x) = e^{\rho x}$, using condition (47), when $\rho$ is small enough, we are in a shallow gradient regime. For linear signal $S(x) = ax + b$, using condition (47), when $a$ is small enough, we are in a shallow gradient regime.

Using the notations of Equations (13)-(14),

$$A_0 \;=\; 0, \quad A_1 \;=\; Npq\,(q-1)\,, \quad B_0 \;=\; Npq\,(q-1)\,\frac{S'\,(K_A-K_I)}{(K_A+S)\,(K_I+S)}.$$

In order to derive an advection-diffusion approximation using Equation (37), we just need to find the first two terms of the Taylor expansion of $\lambda(w)$ in (42). We do that next.

A simple calculation shows that

$$\lambda(w) \;=\; Rq^H \;+\; \frac{HRq^H}{pq}w + Q(w),$$

where $Q(w)$ is the sum of higher orders of $w$ in the Taylor expansion. Plugging the new values of $a_0$ and $a_1$ into Equation (37), we get the following advection diffusion equation:

$$\frac{\partial n}{\partial t} \;=\; \frac{\partial}{\partial x}\left(D\frac{\partial n}{\partial x} - Vn\right), \tag{48}$$

where

$$D = \frac{\nu^2}{2Rq^H}, \quad \text{and} \quad V(x) = \frac{(K_A-K_I)\,S'(x)}{(K_A+S(x))\,(K_I+S(x))}V_0$$

with

$$V_0 = \frac{NH\,(1-q)\,\nu^2}{Npq\,(1-q) + 2Rq^H}.$$

**Modifications for *B. subtilis***

It is known that the activity of *B. subtilis* chemotatic receptors increases in the presence of attractants. This means, in effect, that the roles of $K_I$ and $K_A$ are inverted in the formula for activity: now $K_I > K_A$. Furthermore, tumbling (due to CW rotation of flagella) is induced by lack of activity, which we may model by replacing $a$ by the fraction of inactive receptors, $1 - a$, in the simplified *E. coli* model considered earlier.

Thus, we now assume that the internal state evolves according to the following ODE system:

$$\frac{dy}{dt} \;=\; py(q-a), \tag{49}$$

where we use the following form for activity:

$$a = \frac{1}{1 + K\left(\dfrac{S+K_I}{(S+K_A)\,y}\right)^N}$$

and $p$, $q$, $K$, and $N$, $K_I$, and $K_A$ are positive constants, where now $K_I > K_A$. Recall that $q$ is between zero and one.

We assume now the following form for the tumbling rate:

$$\lambda(y,S) \;=\; R(A-a)^H, \tag{50}$$

where $A$ and $R$ are positive constants. (We assume that $A > q$, which is the case if $A = 1$.)

The objective is to derive a parabolic equation for the macroscopic density function.

As in the previous example, let $w \;=\; p(a-q)$. Then, a simple calculation shows that

$$\begin{aligned} \frac{dw}{dt} \;&=\; \frac{N}{p}(w+pq)(w+pq-p)\left(w \pm \frac{\nu S'\,(K_A-K_I)}{(K_A+S)\,(K_I+S)}\right) \\ \lambda(w) \;&=\; \frac{R}{p^H}(pA-pq-w)^H. \end{aligned} \tag{51}$$

Since $\frac{dw}{dt}$ is exactly the same as in Example , we get the same expressions for $A_i$'s and $B_i$'s, namely:

$$A_0 \;=\; 0, \quad A_1 \;=\; Npq\,(q-1)\,, \quad B_0 \;=\; Npq\,(q-1)\,\frac{S'\,(K_A-K_I)}{(K_A+S)\,(K_I+S)}. \tag{52}$$

In order to derive an advection-diffusion approximation using Equation (37), we just need to find the first two terms of the Taylor expansion of $\lambda(w)$. We do that next.

A simple calculation shows that

$$\lambda(w) \;=\; R(A-q)^H - \frac{RH}{p}(A-q)^{H-1}w + Q(w),$$

where $Q(w)$ is the sum of higher orders of $w$ in the Taylor expansion. Plugging the new values of $a_0$ and $a_1$ into Equation (37), we get the following advection diffusion equation:

$$\frac{\partial n}{\partial t} \;=\; \frac{\partial}{\partial x}\left(D\frac{\partial n}{\partial x} - Vn\right), \tag{53}$$

where

$$D \;=\; \frac{\nu^2}{2R(A-q)^H}, \quad \text{and} \quad V \;=\; \frac{(K_I - K_A)S'(x)}{(K_A+S)(K_I+S)}V_0,$$

with

$$V_0 \;=\; \left(\frac{1-q}{A-q}\right)\frac{NqH\nu^2}{Npq(1-q)+2R(1-q)^H},$$

that can be also rearranged in a more compact form, gives us Equation (2) as presented in the main text:

$$V_C = \frac{\chi_0}{(K_1+C)(K_2+C)}C' \tag{54}$$

with

$$V_C = V, K_1 = K_I, K_2 = K_A, C = S\chi_0 = V_0(K_I - K_A).$$

Thus, a formula of exactly the same form as for *E. coli* has been obtained.

**Our mathematical model best captures aerotaxis in B. subtilis** In order to assess how the model we propose compares to alternative solutions in literature we grouped previous advection-diffusion chemotaxis models in 3 main classes: KS, LS and RTBL models (see following section). Each of these models has a different expression of the chemotactic speed $V_C$ and they range from fully phenomenological (e.g. KS) to biophysically-informed approaches (like RTBL). The vast majority of the other advection-diffusion models used to capture chemotaxis can be derived from the ones we consider in the following.

We compared the performance of the models by plotting the prediction (Fig. S3-S9) of the best combination of parameters the optimization algorithm found over 100 iterations and its prediction error (Fig. 5, see Eq. 5 in the main text). For each model we also plot the distribution of prediction errors of the 100 solutions to the optimization problem.

Notably, for the KS model the genetic algorithm consistently identified a single solution to the parameter optimization problem (Fig. S3), hence the tight distribution in Fig. 5. Similar results in terms of prediction accuracy (and therefore SSE, see Fig. 5) can be achieved using the best solution identified for the LS model (Fig. S4). A significant improvement, instead, can be achieved using

the RTBL model (Fig. S5 and Fig. 5): the best parameter set found in this case achieves an SSE significantly smaller than in the previous cases ($0.95 \cdot 10^{-1}$ compared to $1.84 \cdot 10^{-1}$ for the LS and $1.90 \cdot 10^{-1}$ for the KS models). However the model we propose displays the smallest prediction error ($0.73 \cdot 10^{-1}$, Fig. 5) and therefore best captures the body of experimental data we describe (Fig. 1C).

## 1 SI Materials and Methods

### Growth protocol

B. subtilis strain OI1085 cells from a frozen (-80°C) stock were resuspended in 2 mL of Cap Assay Minimal media (50 mM KH2PO4, 50 mM K2HPO4, 1 mM MgCl2, 1 mM NH4SO4, 0.14 mM CaCl2, 0.01 mM MnCl2, 0.20 mM MgCl2), adding 15 $\mu$L HMT (5 mg/mL each of histidine, methionine, and tryptophan, filter sterilized), 50 $mu$L Tryptone Broth (10 g Tryptone (Difco) and 5 g NaCl in 1 L of distilled water), and 50 $\mu$L 1 M Sorbitol (filter sterilized). The culture was incubated at 37°C while shaking at 250 rpm until OD600 = 0.3 was reached. The culture was then diluted 1:10 in fresh media before injection in the microfluidic device, to ensure cells were in sufficiently low abundance to not affect the oxygen gradient via respiration.

## 2 Microfluidic fabrication, experimental operation and image analysis

In order to generate oxygen gradients, the source and sink channels were each connected to a gas-mixing unit, supplied by gas tanks (Air Gas, MA). We used 100% nitrogen as well as 0.1%, 1%, 20% and 100% oxygen/nitrogen mixtures. Each gas-mixing unit was composed of two high-precision flow controllers (Cole Parmer, IL), one for the appropriate mixture of oxygen and the other for nitrogen, controlled by a MATLAB routine to achieve the final oxygen concentration that would be flown into the source or sink channel. The sum of the flow rates in each line was set to 10 mL/min, while the ratio was set to achieve the desired oxygen concentration. The outlets of the two flow controllers in each mixer were connected using a Y-junction, and low oxygen permeability tubing (C-flex Ultra, Cole Parmer, IL) was used to connect all the components to the microfluidic device. To fabricate the microfluidic device we devised a precision cutting strategy based on piezoelectric actuation to remove three 38 mm-long bands from a 200 $\mu$m thick PDMS sheet. This yielded three parallel grooves piercing through the full depth of the PDMS sheet: the central one ('test channel', 460 $\mu$m wide) was separated from each of the flanking ones ('sink channel' and 'source channel') by a 220 $\mu$m thick PDMS wall. We then used a handheld plasma bonder (BD20AC, ETP) to irreversibly bond the PDMS structure to two 2x3 inch glass slides, one at the top and one at the bottom. Inlets and outlets were obtained by drilling holes ($\oslash$=1 mm) in the glass slides before bonding. In a typical experiment, we flowed the desired oxygen mixtures in the source and sink channels and allowed them to diffuse within the device. Of note, the presence of the 220 $\mu$m thick PDMS wall separating the test channel from the sink channel implied that the minimum oxygen concentration in the test channel was higher than the concentration in

the sink channel. Similarly, the maximum concentration in the test channel was lower than the concentration in the source channel. For example, a 0%-100% case (0 M in the sink channel and $\approx 8$ mM, on the other end, at the interface between PDMS and the source channel) corresponds to an oxygen gradient ranging from 4.6% (60 $\mu$M) to 95% (1.24 mM, 100% oxygen in water corresponding to 1.3 mM) in the test channel (see Table 1 in the Supplementary Information). Bacteria were then injected in the test channel and glass coverslips were used to seal its inlet and outlet of the test channel to suppress any residual flow. Cells reached steady state distribution within 5 minutes after the injection (Fig. 4). We then used an automated acquisition routine to capture 30,000 phase-contrast images of the same location along the test channel (equidistant from the inlet and outlet) at 67 ms intervals over 33 min (20 objective; Andor Zyla camera with 6.5 $\mu$m/pixel (leading to 0.33 $\mu$m/pixel resolution); see Materials and Methods). Each image contained 30-80 individual cells, making for (1-3)$\cdot 10^6$ total recorded cell positions and an estimated 380-1020 individual bacteria included in the analysis. From these, we quantified the concentration of bacteria $\mathrm{B}(x)$ in the direction $x$ across the channel, normalized to a mean of 1 for comparison among different conditions (see Materials and Methods; Fig. 1C). The large number of bacterial positions recorded in each experiment enabled the quantification of $\mathrm{B}(x)$ with a spatial ($x$) resolution of 4.6 $\mu$m and minimal noise (Fig. 1B,C), which proved fundamental for robust model identification. We imaged the bacteria at channel mid-depth using an inverted microscope (Eclipse TE2000-E; Nikon) with a 20 phase-contrast objective (NA = 0.45) and an sCMOS camera (Andor Zyla). A custom MATLAB (Mathworks, MA) algorithm was used for image analysis to accurately identify individual cell coordinates. The normalized bacterial concentration, $\mathrm{B}(x)$, was obtained from the histogram

of the number of bacteria in one hundred bins along the x direction, each 4.6 $\mu$m wide and together covering the 460 $\mu$m width of the test channel, and then normalizing this distribution to a mean of 1. The uncertainty in the estimate of $\mathrm{B}(x)$ was obtained via bootstrapping bacterial x coordinates from all the experiments available for each of the 33 gradients were pooled together. One million samples of 10,000 coordinates each were then analyzed for each gradient to obtain an equivalent number of estimates of $\mathrm{B}(x)$. The extents of the shaded area in Fig. 1C are obtained as the average $\mathrm{B}(x)$ plus/minus its standard deviation calculated over $10^6$ $\mathrm{B}(x)$ bootstrapped profiles.

**Derivation and identification of the mathematical model**

Starting from a Fokker-Planck approximation of the motion of B. subtilis in an oxygen gradient (Supplementary Information) we derived the expression of $V_C$ reported in Eq. 2 in the main text. In order to fully characterize the model we need to identify each of its three parameters $K_1$, $K_2$ and $\chi_0$ - note that $D_B$ is measured experimentally (see Supplementary Information and Fig. S2). To this aim we developed a genetic-algorithm-based multi-experimental fitting procedure designed to find the combination of parameter values that minimized the sum of the squared errors between model predictions and experimental data

$$\mathrm{SSE}(K_1, K_2, \chi_0) = \sum_{i=1}^{n} \frac{\sqrt{(B_E(x) - B_S(x))^2 \cdot w(x)}}{n} \tag{55}$$

where $n = 33$ is the number of experimental designs, $w(x)$ is a vector of weights increasing

linearly from 1 to 1000 (empirically found to ensure the best results in terms of prediction error were attained), $B_E(x)$ are the experimental data and $B_S(x)$ the simulated accumulation profiles via numerical integration ($\Delta x = 10$ nm) of:

$$B(x) = \frac{e^{\frac{\chi_0 \nabla C}{D_B} \int_0^x f(\xi) d\xi}}{\int_0^W f(\xi) d\xi} \tag{56}$$

with test channel width $W = 460\ \mu m$ and $f(\xi) = 1/((K1 + C(\xi))(K2 + C(\xi)))$ for the model in Eq. 2 in the main text. This expression of $\mathrm{B}(x)$ can be obtained plugging Eq. 2 in Eq. 1 in the main text, using the linearity of the oxygen gradient (i.e. $\nabla C$ independent of $\xi$) and posing $\frac{\partial B}{\partial t} = 0$. At each iteration the genetic algorithm generated a number of random solutions, ranked them based on Eq. 55, the worst solutions, selected the best ones and applied "cross-over" and "mutation" to obtain new solutions to be evaluated at the next iteration [50]. The search for a solution stopped when a stall was detected, i.e., when the average change in $\mathrm{SSE}(\chi_0, K_1, K_2)$ over 50 iterations was smaller than $10^{-6}$. The reported parameter set is the best combination identified over 100 repetitions of this procedure. We adopted the same method to identify the parameter values for all models (see Supplementary Information).

**Robustness analysis of parameter estimates**

Although very powerful at solving complex optimization problems, Genetic Algorithms do not provide any guarantee of convergence. As a consequence of this, a set of "optimal parameters"

obtained as a result of the optimization, might actually be a local, rather than a global solution - these are solutions that optimize the objective function in a sufficiently large neighborhood of, but not the entire, space of parameters. Yet, at the end of the parameter optimization process we would ideally identify a set of values that minimizes the cost function (Eq. S62) globally rather than locally.

To assess whether the values obtained from the Genetic Algorithm could be outcompeted by other combinations of values, we decided to adopt a Naive Grid Search approach. The principle behind this method is simple: the set of values each parameter can take is discretized and all the combination of discretized parameters are evaluated using the cost function. The more fine-grained the discretization is, the more this approach resembles an exhaustive search. The main limitation of this approach is that for large numbers of parameters and/or parameter values the number of objective function evaluations quickly increases and ultimately makes the problem intractable.

As customary in these cases, we assigned to each parameter identification task (i.e., each model among the ones we considered) a budget of "function evaluations" equal to $10^5$. For each of the $i$ parameters in that model, we identified a physically feasible set of values, and discretized it into $M$ values, with $M$ being the closest integer to $10^{\frac{5}{i}}$. We then evaluated the cost function for each of these combinations and, for each model, the value of the minimum cost identified by the Naive Grid Search method was compared to the minimum found by the Genetic Algorithm (Fig. S10).

For both the KS and the LS models (1 and 2 parameters, respectively) we confirmed that the Naive Grid Search identified values of the optimal parameters substantially undistinguishable from the ones returned by the Genetic Algorithm. For the RTBL and the Finite Range Log-sensing regime,

instead, the Naive Grid Search algorithm returned values different from the Genetic Algorithm and, in both cases, characterized by higher value the cost function - suggesting that the parameter values identified by the Naive Grid Search are not global optima. These results indicate that it is unlikely that the parameter sets identified by the Genetic Algorithm for our model represent local optima and that they are instead the global optima we sought.

### Model validation on transient aerotaxis

As a stringent validation of the model, we tested its performance in predicting the population migration in a transient aerotaxis experiment. At the start of the experiment, sink and source channels both contained a flow of 21% oxygen and cells were allowed time to equilibrate to their steady state distribution, which was uniform given the uniform oxygen concentration (Fig. 4). At time zero we started flowing 0% and 0.05% oxygen in the sink and source channels, respectively, and recorded the spatial distribution of bacteria across the test channel at 100 frames/s for 4 min To produce $\mathrm{B}(x)$, we binned 200 frames (2 s) in one time point, in order to minimize noise. The model prediction was obtained by integrating Eqs. 2 and 1 numerically with COMSOL (Comsol Inc., MA). We modeled oxygen dynamics using the diffusion equation and representing the microfluidic device as a one-dimensional domain with three parts: the 460 $\mu$m wide test channel (460 $\mu$m wide) and the two, 220 $\mu$m wide, flanking PDMS walls, at the outer end of which the experimentally imposed source and sink oxygen concentrations were prescribed. We note that, given the relative composition of the Cap Assay Minimal medium (essentially water supplemented with very small quantities of salts, amino acids and sorbitol) and, coherently with what has been previously reported[16], we

approximated the growth medium as water for the purpose of our simulations; therefore we set the diffusion coefficient of oxygen in water to $2 \cdot 10^{-9}$ $m^2$/s. We observe that: (a) temperature fluctuations have been ignored here as all the experiments have been carried out under temperature control, (b) the density of bacteria was low enough[10] ($OD_{600}$=0.03) to allow us to ignore the effect of respiration on the gradient and (c) although we do not expect inhomogeneity to be introduced in the PDMS matrix as part of the microfabrication process, we did not assess how any residual heterogeneities would have affected the diffusion dynamics. Oxygen profiles obtained as a result of the simulations were then used as input in the bacterial transport equation (Eqs. 1 and 2), which was solved in the test channel with a time step of 0.1 s and a spatial resolution of 4.6 $\mu$m, after ensuring these choices were sufficient to have a converged solution. The models used in our comparative analysis are introduced and discussed in this section.

**KS model**

Developed in the early 70s by Keller and Segel [41], this was the first mathematical model that aimed at quantitatively capturing chemotaxis. Studying slime molds the authors observed that chemotaxis is the result of random (diffusion) and directed motility (advection) of microorganisms and consequently decided to use advection-diffusion models to capture it. When it came to the choice of an expression for the advection (i.e. chemotactic) speed, $V_C$, Keller and Segel took a phenomenological approach and assumed it was directly proportional to the chemoattractant gradient $\nabla C$ (rescaled by a constant $\chi_0$) and inversely proportional to $C$ the chemoattractant concentration:

$$V_C = \chi_0 \frac{\nabla C}{C}$$

This expression of $V_C$ has a singularity for $C = 0$ M, i.e. bacteria achieve higher and higher chemotactic speed as the concentration of the chemoattractant decreases; a rather counterintuitive result. However, in a follow-up paper [44], Keller and Segel noted this specific choice of the functional form of $V_C$ allowed their model to predict band-formation: a phenomenon observed few years back in *in-vivo* experiments carried out by Adler [45]. A preliminary confirmation of the validity of this model came few years later when Holz and Chen [46] demonstrated it was able to predict *E. coli*'s response to serine gradients.

Interestingly, even before Dahlquist and co-workers first suggested bacteria responded to relative, rather than absolute, changes in chemical concentrations [47], the KS model supported logarithmic sensing at all concentrations ($V_C \propto \nabla C/C \; \forall C$). Recent studies highlighted that logarithmic sensing, and therefore gradient rescaling, only takes place over a finite interval of concentrations [48], pointing at a fundamental limitation of the KS model.

The need to provide biological ground to the proposed models then emerged and triggered a quest for a more "biologically realistic" alternative [40] to the original KS model; this is the direction Lapidus and Schiller took while developing their model (LS) [51].

To test the ability of this model to recapitulate our experimental results, and compare its prediction capabilities with other models, we adopted the same approach reported in the main text for the

"finite-regime log-sensing" model we propose (see "Derivation and Identification of the Mathematical Model"). We ran 100 instances of a genetic algorithm meant to identify the value of $\chi_0$ (the only free parameter in this model) that minimizes the average mismatch between model prediction and experimental results over the whole dataset. It should be noted that, based on Eq. (4) (see main text) and assuming $D_B$ does not depend on space, the steady state distribution of bacteria $B(x)$ can be rewritten as:

$$B(x) = \frac{e^{\frac{\chi_0 \nabla C}{D_B} \cdot \int_0^x \frac{1}{C(\xi)} d\xi}}{\int_0^W \frac{1}{C(\xi)} d\xi} \tag{57}$$

where we observe that $\chi_0$ and $D_B$ in this model are "structurally unidentifiable". Given the physical meaning of the $\chi_0$ and $D_B$ we imposed a non negativity constraint on the optimization problem meant to identify the value of $\chi_0/D_B$, collected the results of the optimization procedures and plotted the prediction of the model achieving the best accuracy ($\chi_0/D_B = 2.66\ \mu m$, Fig. S3).

**LS model**

Motivated by the mismatch between model predictions of the original KS formulation [52–54] and the experiments reported in [47], Lapidus and Schiller set out to propose a functional form of the chemotactic speed that incorporated one of the most relevant biochemical properties of chemoreceptors: the dissociation constant between the ligand and the receptor itself.

They succeeded in this effort and proposed a formulation of $V_C$ directly proportional to the chemo-

tactic sensitivity coefficient $\chi_0$ and inversely proportional to the squared sum of $K$ and the chemoattractant concentration $C$:

$$V_C = \chi_0 \frac{\nabla C}{(K + C)^2}$$

By using population scale measurements of bacterial fluxes, not only were Lapidus and Schilller able to identify the values of $\chi_0$ and $K$, they also showed the predictions of their model were in good agreement with the experimental results.

It is worth noting that, while achieving good performance in capturing the experimental results in [51], the LS model does not support logarithmic sensing. Moreover, as our understanding of the cascade of signaling events leading to chemotaxis furthered, an increasing number of approaches focused on bridging single cell behavior and population level phenomena.

To assess the ability of this model to capture our data we followed the same approach described for the KS model. In this case, however, the parameters to be identified are both $\chi_0/D_B$ and $K$. We set non-negativity constraints for this identification task following the same line of reasoning mentioned above and recorded the results of the 100 optimization procedures. The solutions to the optimization problem is plotted in Fig. S4 ($\chi_0/D_B = 57.89$ and $K = 1.39 \cdot 10^{-5}$ M).

**RTBL model**

The RTBL model, developed by Rivero and colleagues [43], achieves a macroscopic characterization of bacterial chemotaxis using microscopic variables involved in the chemotactic response of single cells (e.g. receptor occupation and swimming speed). In order to derive their model Rivero and colleagues considered two sub-populations of bacteria ($p^+$ and $p^-$) exposed to a chemoattractant gradient in a 1D domain. Each bacterium can either proceed from left to right or viceversa; this will determine which subpopulation it belongs to. Tumbling makes a bacterium switch from one group to the other; just like we would expect to happen *in-vivo*, the probability of tumbling depends on the time derivative of the number of bound receptors. In this framework, following the steps reported in Appendix A in [55], one can derive the expression of the chemotactic speed $V_C$:

$$V_C = \frac{2}{3} V \tanh\left(\frac{\chi_0}{2V}\frac{\nabla C}{(K+C)^2}\right)$$

where $V$ is the swimming speed of bacteria.

While being one of the most advanced results in chemotaxis, this model does not recapitulate the most recent observations [56] regarding logarithmic sensing and Fold Change Detection in *E. coli*'s chemotaxis.

Consistently with what we previously reported, we probed the ability of the RTBL model to capture our dataset running 100 instances of our optimization procedure. In this case the parameters to be identified were three: $\chi_0$, $K$ and $V$. For all of them we set non-negativity constraints, following

the considerations we previously discussed; moreover we restricted $V$, the swimming speed, to not exceed 40 $\mu m/s$ (we set this constraint according to experimental quantification of bacterial swimming speed we obtained while measuring $D_B$). Also in this case we collected statistics on the prediction error of the solutions identified during the 100 runs of the optimization procedure (Fig. 5) and we plotted the results from the simulation of the best among the 100 solutions identified by the genetic algorithm in Fig. S5 ($\chi_0 = 7.10 \cdot 10^{-8}$ $\mathrm{m}^2/\mathrm{s}$, $K = 7.01 \cdot 10^{-6}$ M and $V = 39.4 \cdot 10^{-5}$ m/s).

## References


33. J.R. Howse,R.A.L. Jones, A.J. Ryan,T. Gough, R. Vafabakhsh, and R. Golestanian, *Self-Motile Colloidal Particles: From Directed Propulsion to Random Walk* Physical Review Letters, 99. doi:10.1103/PhysRevLett.99.048102 (2007).

34. A. Sokolov, and I.S. Aranson,*Physical properties of collective motion in suspensions of bacteria* Physical Review Letters, 109. doi:10.1103/PhysRevLett.109.248109 (2012).

35. D.S. Bischoff, and G.W. Ordal *Bacillus subtilis chemotaxis: a deviation from the Escherichia coli paradigm*. Molecular Microbiology, 6(1), 2328. doi:10.1111/j.1365-2958.1992.tb00833.x

36. Othmer H. G., Dunbar S. R., and Alt W. Models of dispersal in biological systems. *J Math Biol*, 26(3):263–98, 1988.

37. L. Jiang, Q. Ouyang, and Y. Tu. Quantitative modeling of escherichia coli chemotactic quantitative modeling of escherichia coli chemotactic motion in environments varying in space and

time. *PLoS Computational Biology*, 6(4):e1000735, 2010.

38. J. Monod, J. Wyman, and J. P. Changeux. On the nature of allosteric transitions: a plausible model. *J. Mol. Biol.*, 12:88–118, May 1965.

39. Y. Tu, T. S. Shimizu, and H. C. Berg. Modeling the chemotactic response of Escherichia coli to time-varying stimuli. *Proc. Natl. Acad. Sci. U.S.A.*, 105:14855–14860, 2008.

40. M. Tindall, P. Maini,S. Porter, and J. Armitage *Overview of mathematical approaches used to model bacterial chemotaxis II: bacterial populations*, Bulletin of Mathematical Biology, 70(6), 15701607 (2008).

41. E. Keller, and L. Segel *Model for chemotaxis*, J. Theor. Biol. 30(2), 225234 (1971).

42. M.A. Rivero-Hudec, and D. Lauffenburger *Quantification of bacterial chemotaxis by measurement of model parameters using the capillary assay* Biotech. Bioeng. 28, 11781190 (1986).

43. M.A. Rivero, R.T. Tranquillo, H.M. Buettner, and D.A. Lauffenburger *Transport models for chemotactic cell-populations based on individual cell behavior* Chem. Eng. Sci. 44:28812897 (1989).

44. E. Keller,and L. Segel, *Traveling bands of chemotactic bacteria: a theoretical analysis* Journal of Theoretical Biology. 30(2), 235248 (1971).

45. J. Adler, *Chemotaxis in bacteria* Science 153, 708716 (1966).

46. M. Holz, and S.H. Chen, *Spatio-temporal structure of migrating chemotactic band of Escherichia coli. I. Traveling band profile* Biophysical Journal, 26, 243261 (1979).

47. F.W. Dahlquist, P. Lovely and D.E. Koshland *Quantitative analysis of bacterial migration in chemotaxis* Nat. New Biol. 1972;236:120123 (1972).

48. Y.V. Kalinin, L. Jiang, Y. Tu, and M. Wu, *Logarithmic sensing in Escherichia coli bacterial chemotaxis*, Biophys. J. 96, 24392448 (2009).

49. T. Scribner,L. Segel and E. Rogers, *A numerical study of the formation and propagation of travelling bands of chemotactic bacteria* J. Theor. Biol. 46, 189219 (1974).

50. Z. Michalewicz,*Genetic Algorithms, Numerical Optimization, and Constraints* Proc. sixth Int. Conf. Genet. algorithms 195, 151158 (1995).

51. R. Lapidus and R. Schiller, *Model for the chemotactic response of a bacterial population* Biophys. J. 16, 779789 (1976).

52. Nossal, R.,Weis, G., 1973. Analysis of a densitometry assay for bacterial chemotaxis. J. Theor. Biol. 41(1), 143147.

53. Segel, L., Jackson, L., 1973. Theoretical analysis of chemotactic movements in bacteria. J. Mechanochem. Cell Motility 2, 2534.

54. Lapidus, R., Schiller, R., 1974. A mathematical model for bacterial chemotaxis. Biophys. J. 14, 825834.

55. Ahmed, T., and Stocker, R. (2008). Experimental verification of the behavioral foundation of bacterial transport parameters using microfluidics. Biophysical Journal, 95, 44814493.

56. Lazova, M. D., Ahmed, T., Bellomo, D., Stocker, R., and Shimizu, T. S. (2011). Response rescaling in bacterial chemotaxis. Proceedings of the National Academy of Sciences of the United States of America, 108, 1387013875.

57. Aminzare, Z., and Sontag, E.D. (2013). Remarks on a population-level model of chemotaxis: advection-diffusion approximation and simulations. arXiv:1302.2605v1.

Table 1: **Oxygen concentrations inside the test channel**. For each oxygen mixture flown in in the sink and source the actual concentrations within the test channel, as well as the number of replicates, are reported here. In each case the bacteria were exposed to a linear gradient with minimum C(0 $\mu m$) and maximum C(460 $\mu m$).

| **Sink [%]** | **Source [%]** | **C(0 $\mu m$) [M]** | **C(460 $\mu m$) [M]** | **Replicates** |
|---|---|---|---|---|
| 0 | 0.01 | 6.04E-09 | 1.24E-07 | 3 |
| 0 | 0.025 | 1.51E-08 | 3.10E-07 | 2 |
| 0 | 0.05 | 3.02E-08 | 6.20E-07 | 7 |
| 0 | 0.075 | 4.53E-08 | 9.30E-07 | 2 |
| 0 | 0.1 | 6.04E-08 | 1.24E-06 | 7 |
| 0 | 0.25 | 1.51E-07 | 3.10E-06 | 4 |
| 0 | 0.5 | 3.02E-07 | 6.20E-06 | 4 |
| 0 | 1 | 6.04E-07 | 1.24E-05 | 5 |
| 0 | 2.5 | 1.51E-06 | 3.10E-05 | 2 |
| 0 | 5 | 3.02E-06 | 6.20E-05 | 4 |
| 0 | 10 | 6.04E-06 | 1.24E-04 | 2 |
| 0 | 20 | 1.21E-05 | 2.48E-04 | 2 |
| 0 | 30 | 1.81E-05 | 3.72E-04 | 2 |
| 0 | 40 | 2.41E-05 | 4.96E-04 | 3 |
| 0 | 50 | 3.02E-05 | 6.20E-04 | 3 |
| 0 | 60 | 3.62E-05 | 7.44E-04 | 2 |
| 0 | 70 | 4.23E-05 | 8.68E-04 | 2 |
| 0 | 80 | 4.83E-05 | 9.92E-04 | 2 |
| 0 | 90 | 5.43E-05 | 1.12E-03 | 3 |
| 0 | 100 | 6.04E-05 | 1.24E-03 | 2 |
| 5 | 10 | 6.80E-05 | 1.27E-04 | 2 |
| 5 | 15 | 7.10E-05 | 1.89E-04 | 3 |
| 10 | 10 | 1.30E-04 | 1.30E-04 | 2 |
| 10 | 15 | 1.33E-04 | 1.92E-04 | 2 |
| 10 | 30 | 1.42E-04 | 3.78E-04 | 2 |
| 10 | 50 | 1.54E-04 | 6.26E-04 | 2 |
| 10 | 70 | 1.66E-04 | 8.74E-04 | 2 |
| 10 | 90 | 1.78E-04 | 1.12E-03 | 2 |
| 15 | 20 | 1.98E-04 | 2.57E-04 | 2 |
| 20 | 20 | 2.60E-04 | 2.60E-04 | 2 |
| 20 | 40 | 2.72E-04 | 5.08E-04 | 2 |
| 20 | 60 | 2.84E-04 | 7.56E-04 | 2 |
| 20 | 80 | 2.96E-04 | 1.00E-03 | 2 |
| 30 | 30 | 3.90E-04 | 3.90E-04 | 2 |
| 30 | 50 | 4.02E-04 | 6.38E-04 | 2 |
| 30 | 70 | 4.14E-04 | 8.86E-04 | 2 |
| 40 | 40 | 5.20E-04 | 5.20E-04 | 2 |
| 50 | 50 | 6.50E-04 | 6.50E-04 | 2 |
| 60 | 60 | 7.80E-04 | 7.80E-04 | 2 |
| 70 | 70 | 9.10E-04 | 9.10E-04 | 2 |
| 80 | 80 | 1.04E-03 | 1.04E-03 | 2 |
| 90 | 90 | 1.17E-03 | 1.17E-03 | 2 |
| 100 | 100 | 1.30E-03 | 1.30E-03 | 2 |

# Supplementary Figures

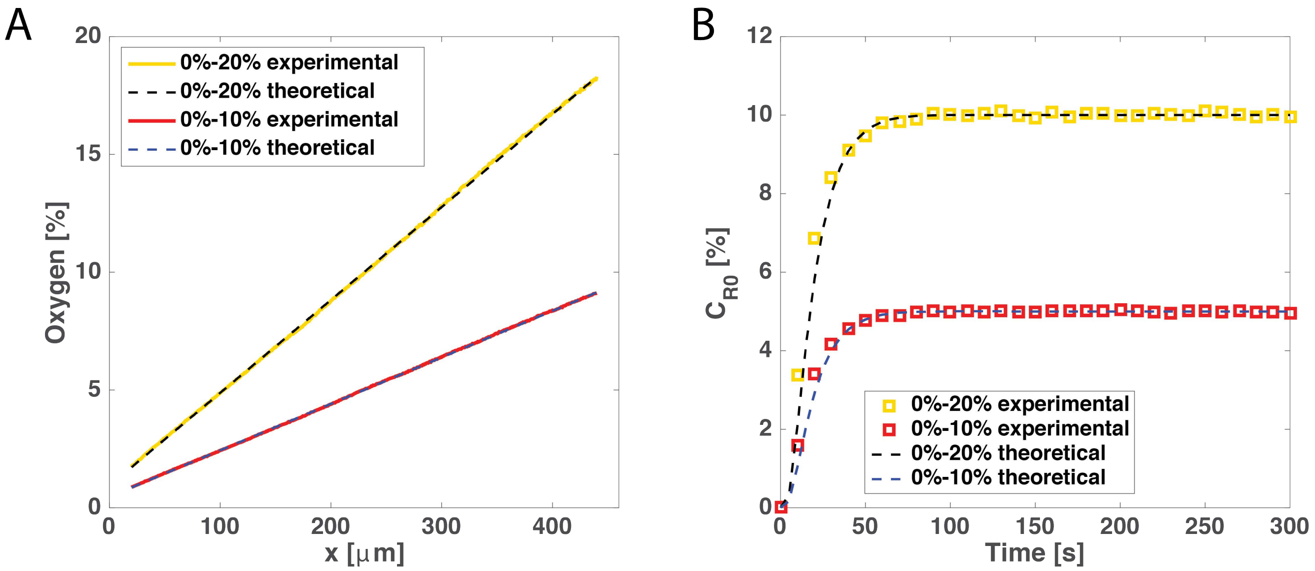


Figure 1: **In-silico and in-vitro analysis of oxygen diffusion in the microfluidic device**. In panel A the steady state oxygen concentration is plotted as a function of space for both the gradients 0%-20% and 0%-10%. Dashed and solid lines represent, respectively, model predictions and experimental quantifications. In panel B $C_{R0}$, the rescaled oxygen concentration at mid-channel is plotted against time: dashed line is model prediction, squares are experimental measurements.

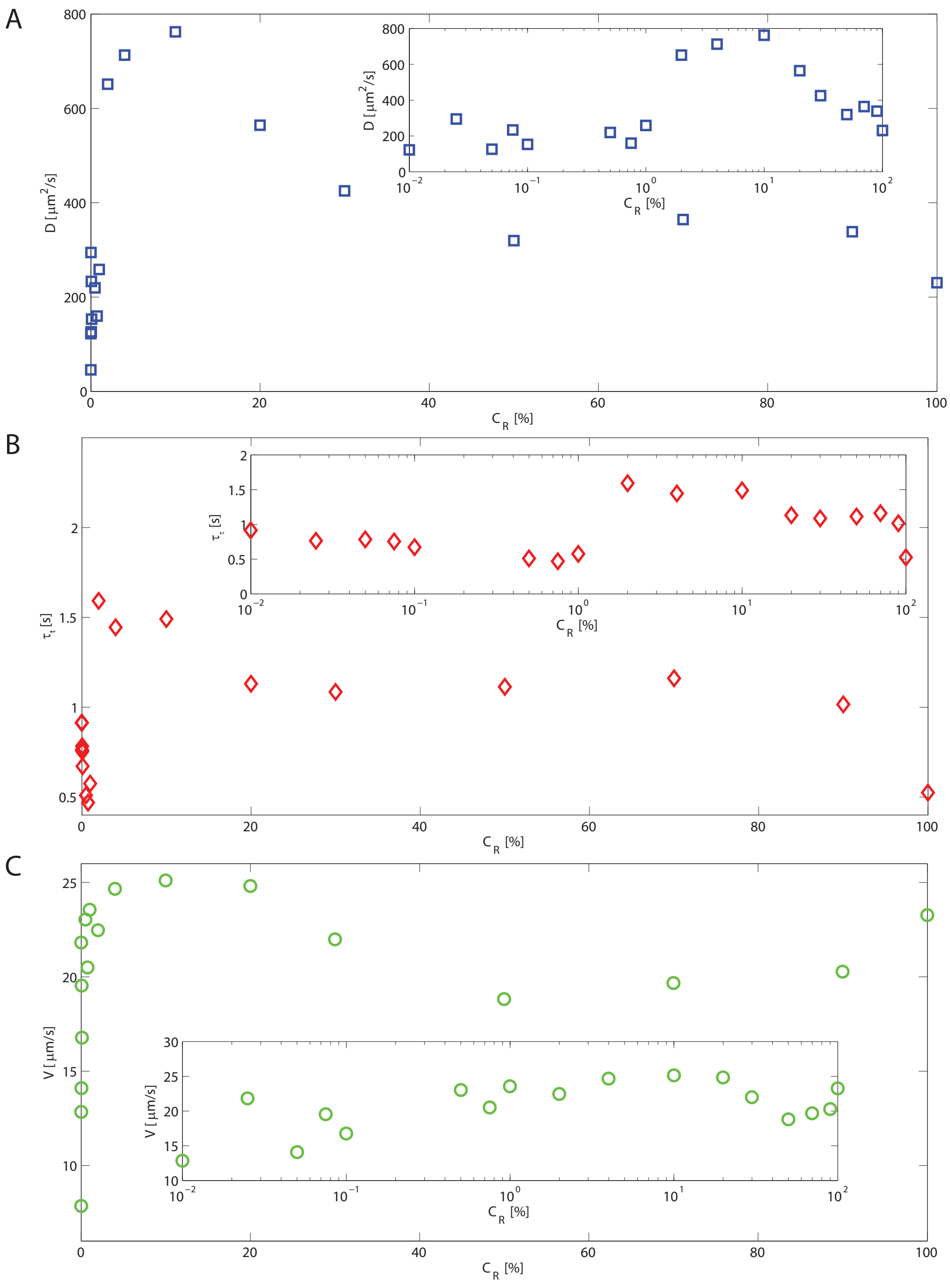


Figure 2: **Quantification of $D_B$.** (A) shows the bacterial diffusion coefficient, $D_B$, plotted against oxygen concentration. (B) and (C) show how the two physical quantities, swimming speed $V$ (measured) and tumble time $\tau_t$ (fitted), contribute to shape $D_B$ (Eq. S1) and their dependence on $O_2$. Semilog plots (inset) illustrate the dependence of these quantities at low oxygen concentrations

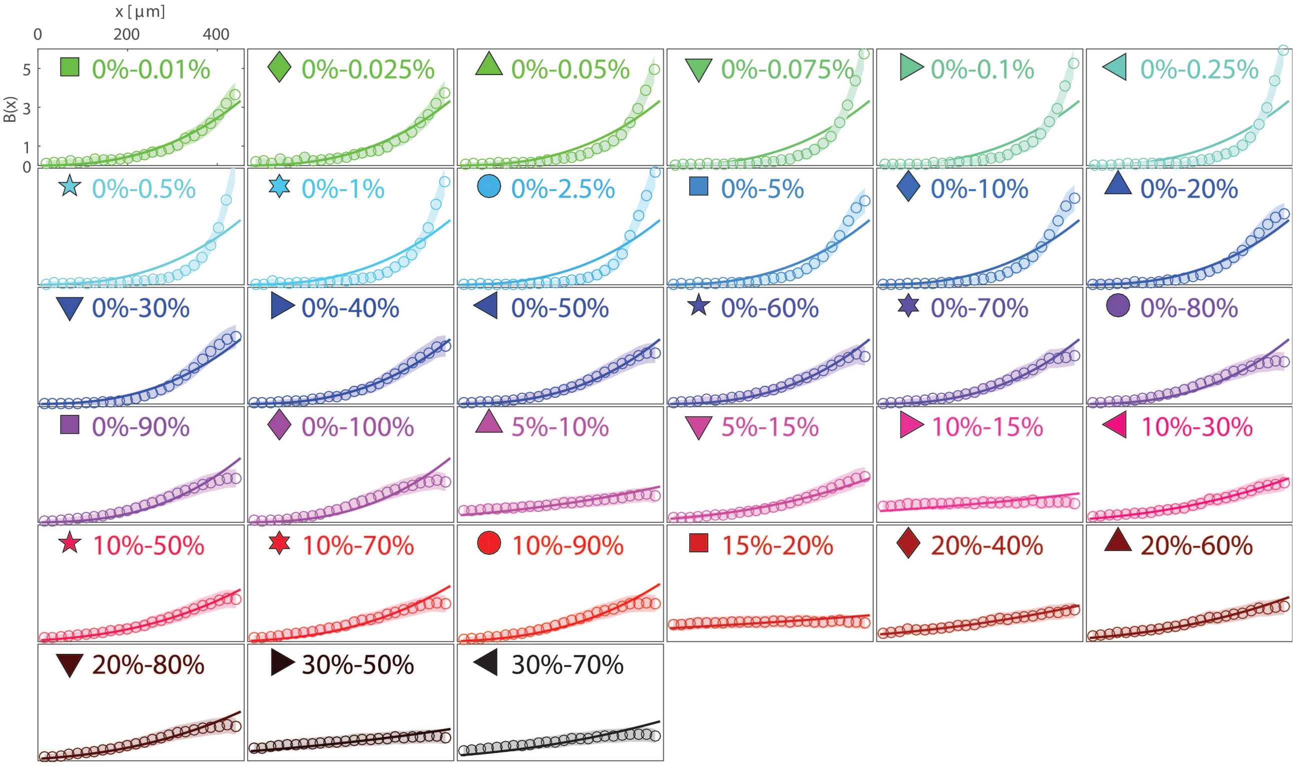


Figure 3: **Best KS model predictions**. Numerical simulation (solid lines) of the KS model with the value of $\chi_0/D$ that minimizes the weighted SSE. Experimental data are represented with circles, shaded area around the them represent $\pm$ standard deviation on the estimates of B(x).

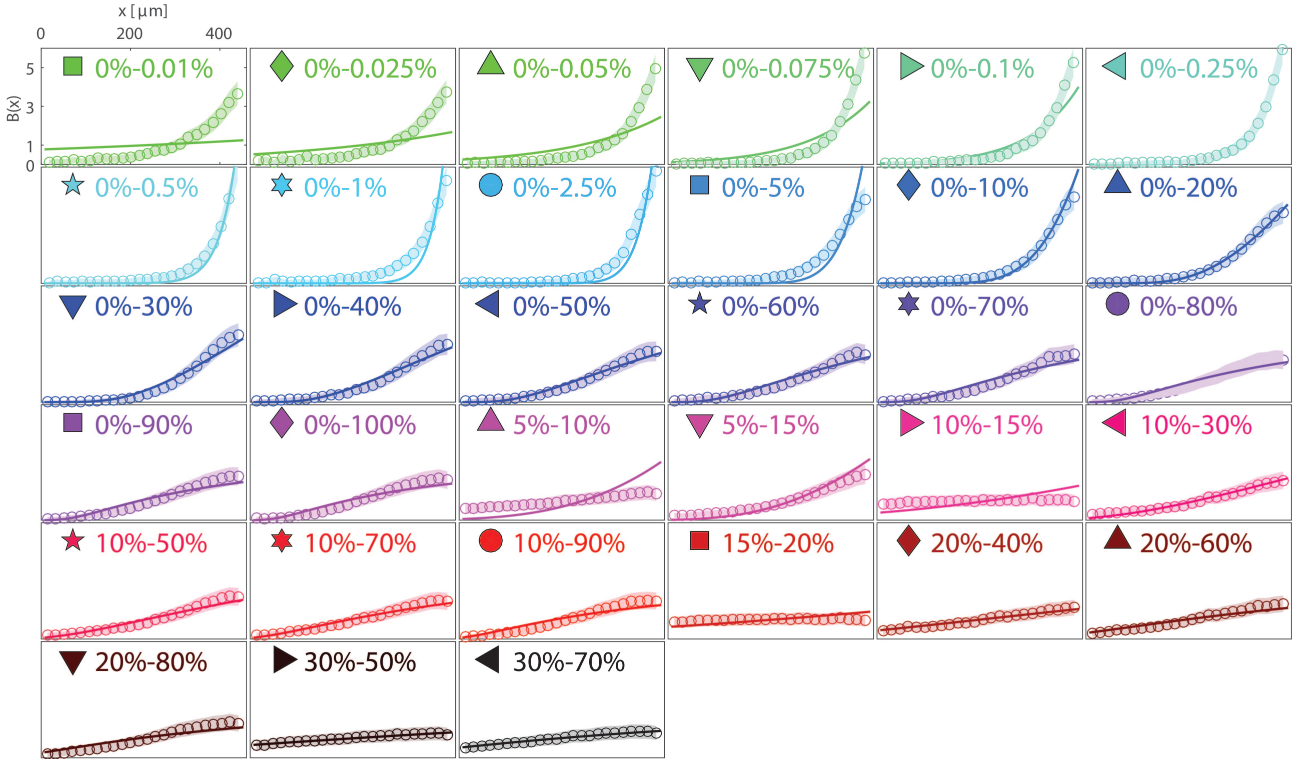


Figure 4: **Best LS model predictions**. Numerical simulation of the RL model with the values of $\chi_0/D$ and $K$ that minimize the weighted SSE. Data are presented as in Fig. S3.

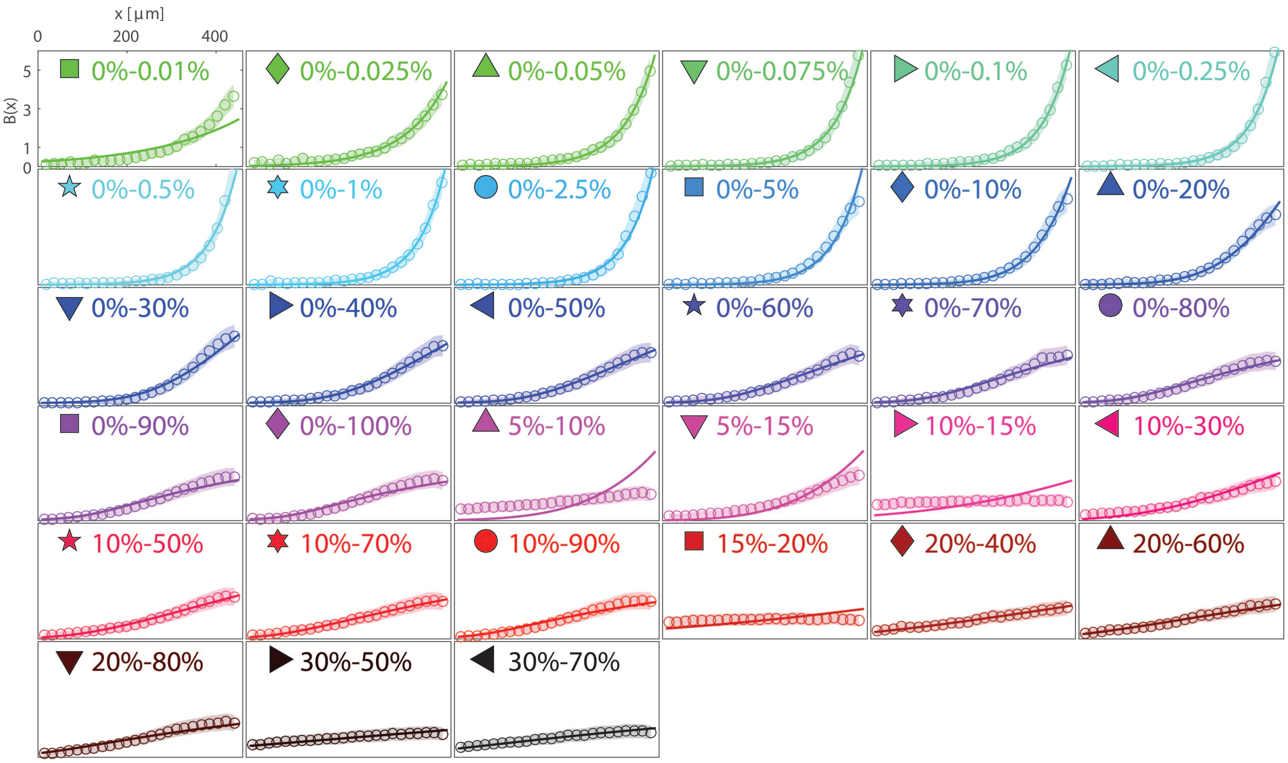


Figure 5: **Best RTBL model predictions**. Numerical simulation of the RTBL model with the values of $v$, $K$ and $\chi_0$ that minimize the weighted SSE. Data are presented as in Fig. S3.

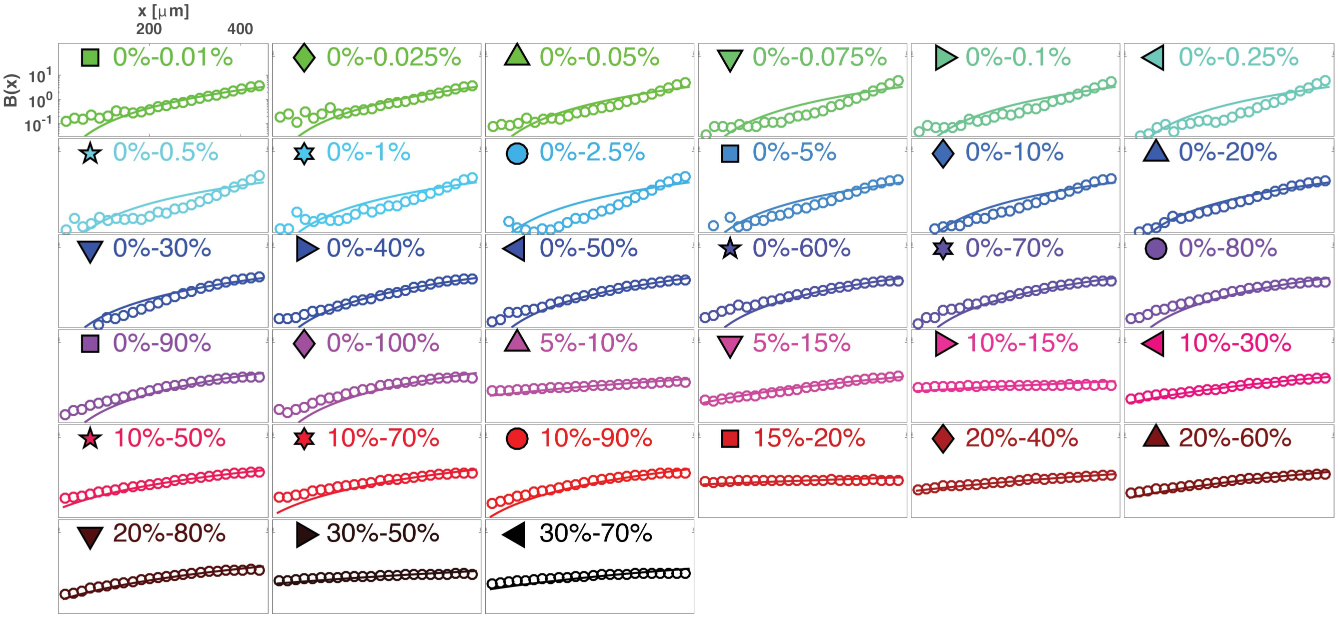


Figure 6: **Best KS model predictions - x axis in log-scale**. The same data presented in Fig. S3 is here presented in log-scale (x axis).

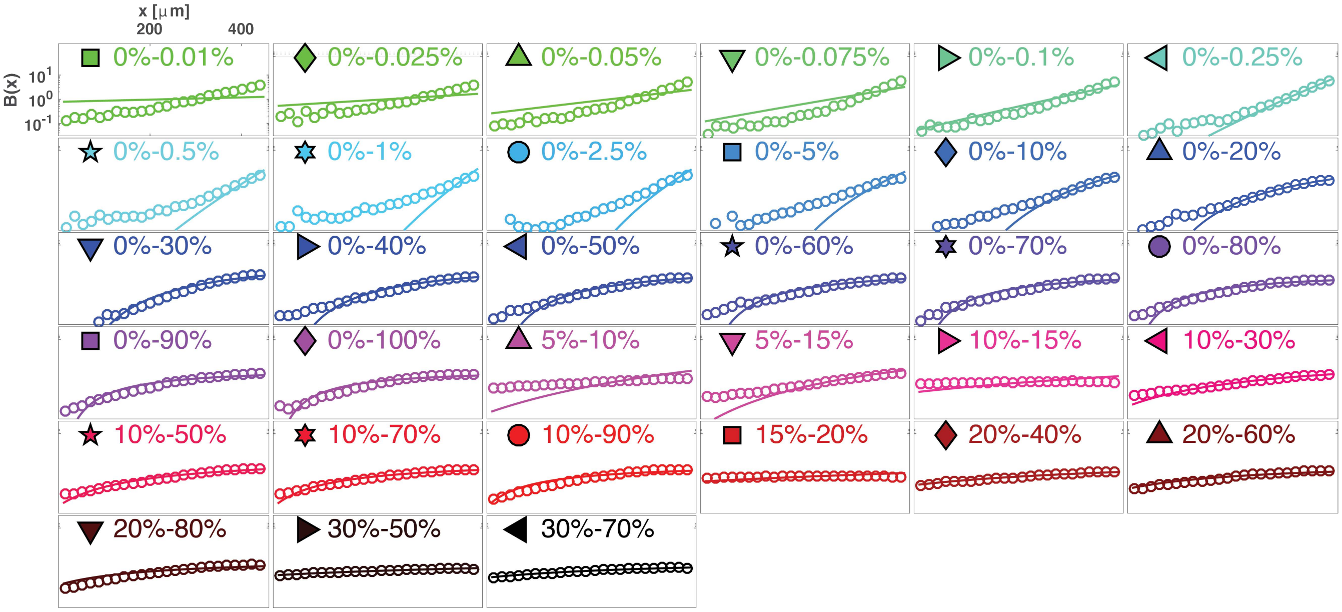


Figure 7: **Best LS model predictions - x axis in log-scale**. The same data presented in Fig. S4 is here presented in log-scale (x axis).

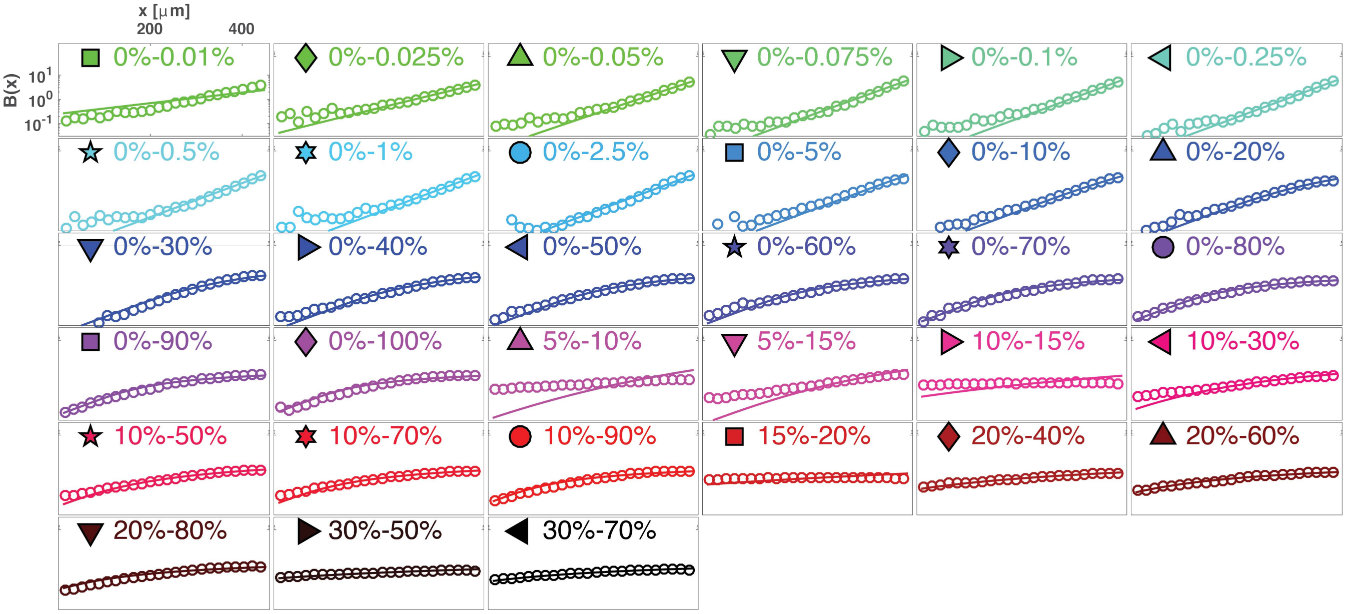


Figure 8: **Best RTBL model predictions - x axis in log-scale**. The same data presented in Fig. S5 is here presented in log-scale (x axis).

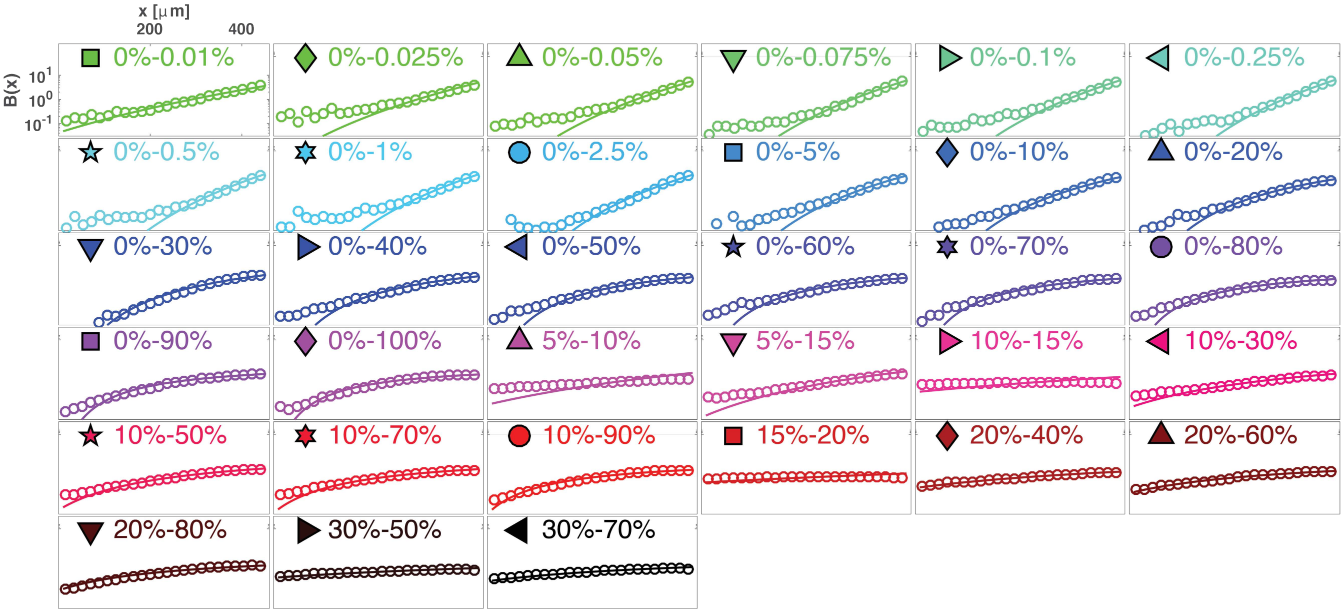


Figure 9: **Best model predictions for the Finite regime log-sensing model - x axis in log-scale**.

The same data presented in Fig. 1 is here presented in log-scale (x axis).

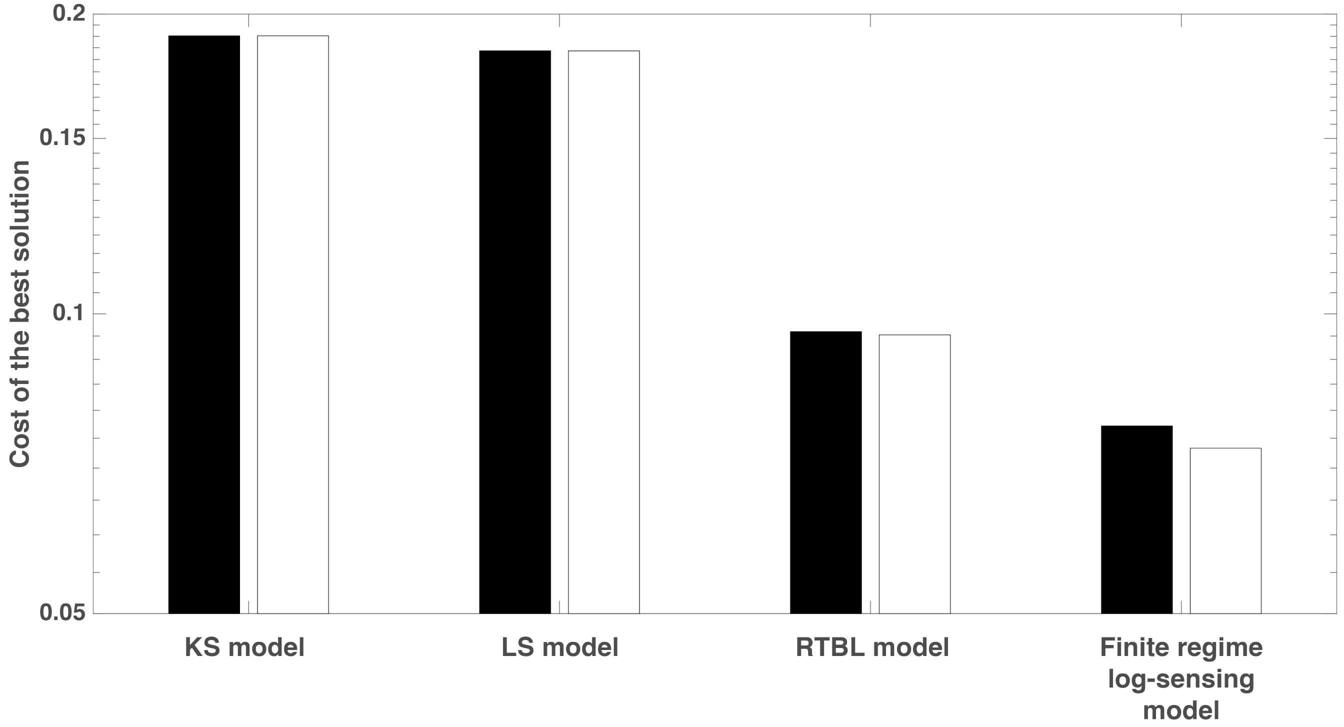


Figure 10: **Comparison of Naive Grid Search and Genetic Algorithm parameter optimization**. The minimum value of the cost function is here plotted for each of the four models as identified by the Naive Grid Search (black bars) and the Genetic Algorithm (white bars). In all the cases the Genetic Algorithm was able to identify a solution that matched or exceeded the quality of the solution identified as best by the Naive Grid Search.

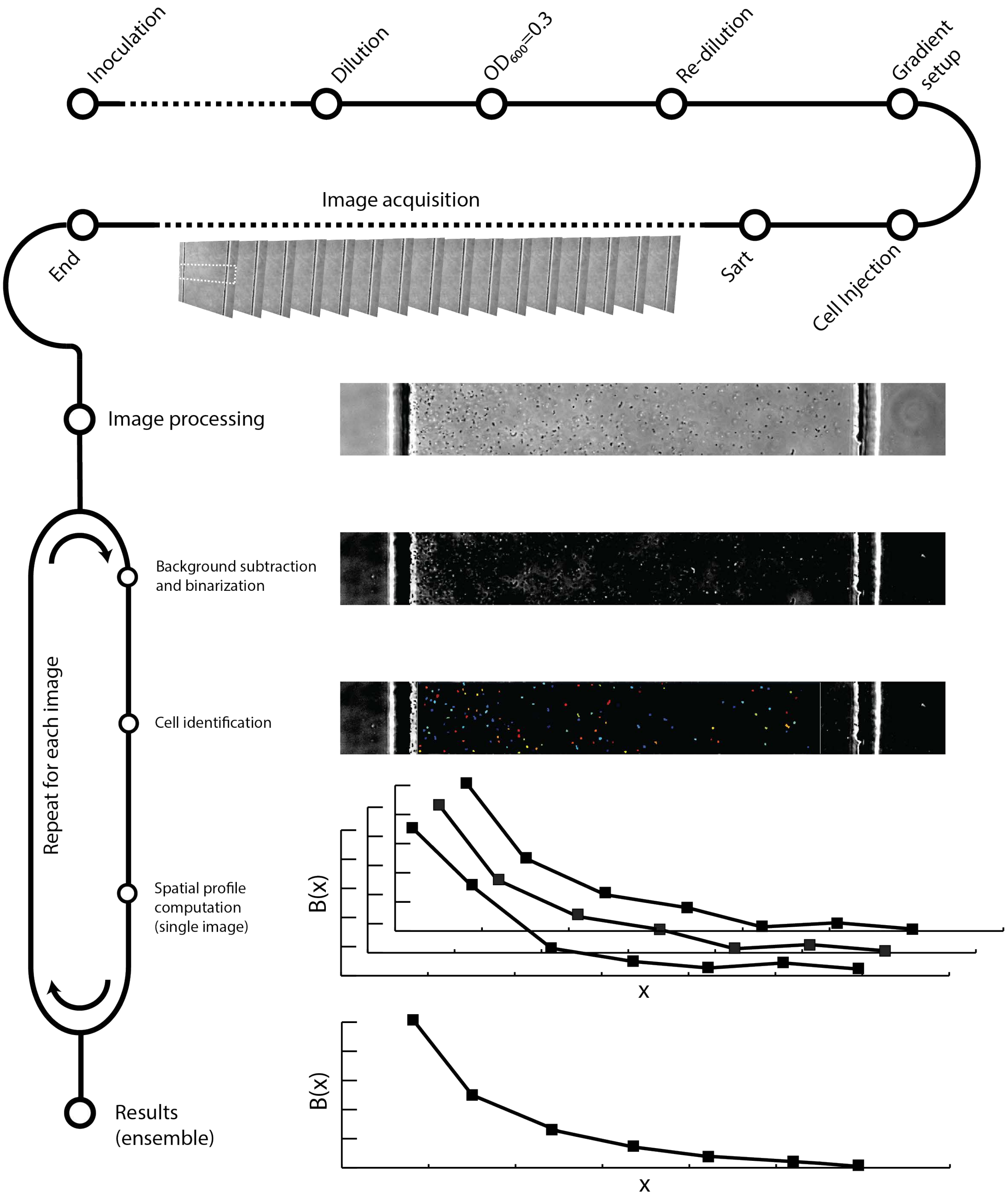


Figure 11: **Experimental setup**. From culturing cells (”Inoculation” to ”Redilution”) to acquiring the microscopy images (“Image acquisition”) and analysing them to extract B(x) for each frame (“Spatial profile computation”), then pooled to compute the final B(x) (“Results”), the sequence of steps of a typical experiment of the type presented in Figure 1C is presented here.

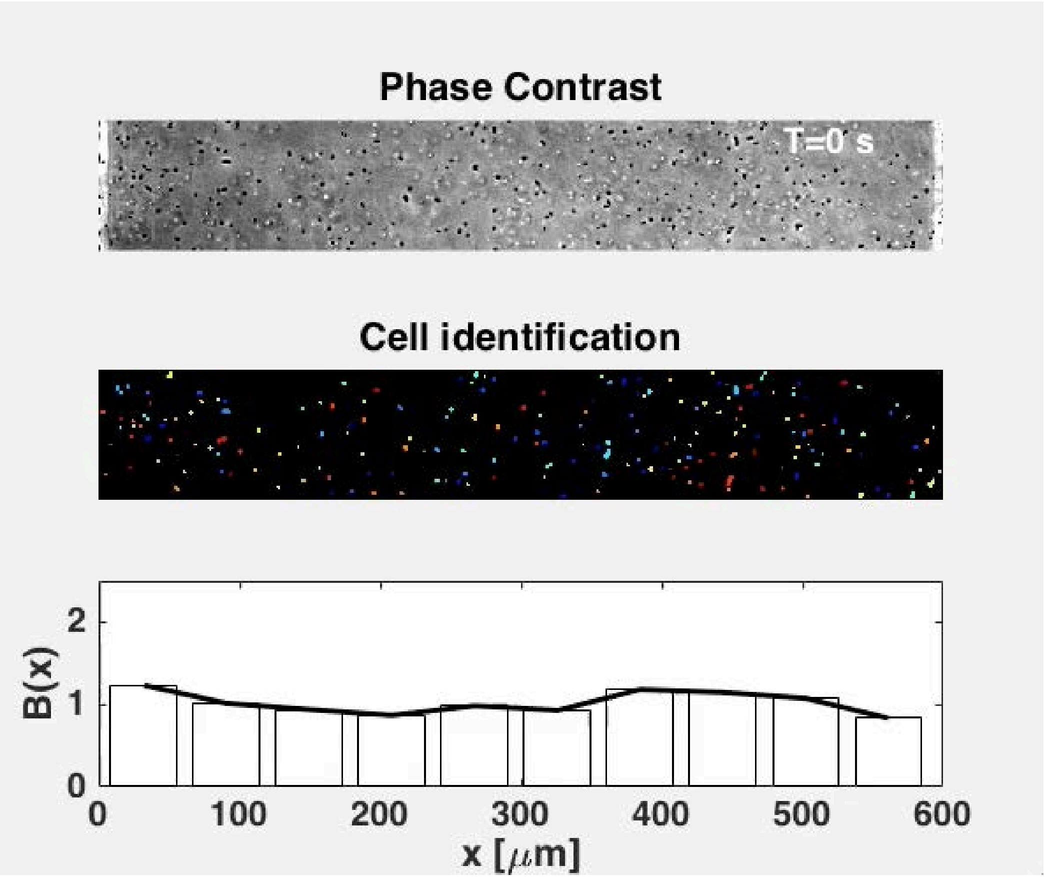


Video S1: **Dynamics of bacteria accumulation**. An example of the dynamics of bacteria accumulation is reported in this video. At time t=0 s the gradient is switched from 20%-20% to 0%-100%; images are acquired at 1 fps in this experiment. Bacteria (black in “Phase contrast”), each identified with a different colour by the image processing processing algorithm (“Cell Identification”), start migrating towards the oxygen rich end (left) until a steady state distribution is reached. In this experiment a concentration of bacteria $\sim$ 3 times higher than usual has been used (to limit the impact of noise, still present, on the computational of the B(x) for each frame, lower panel) as well as a wider test channel (600 $\mu$m in width).
